# Associations Between the Molecular and Optical Properties of Dissolved Organic Matter in the Florida Everglades, a Model Coastal Wetland System

**DOI:** 10.3389/fchem.2015.00066

**Published:** 2015-11-25

**Authors:** Sasha Wagner, Rudolf Jaffé, Kaelin Cawley, Thorsten Dittmar, Aron Stubbins

**Affiliations:** ^1^Marine Sciences Department, Skidaway Institute of Oceanography, The University of GeorgiaSavannah, GA, USA; ^2^Southeast Environmental Research Center, Department of Chemistry and Biochemistry, Florida International UniversityMiami, FL, USA; ^3^Department of Civil, Environmental and Architectural Engineering, Institute of Arctic and Alpine Research, University of Colorado at BoulderBoulder, CO, USA; ^4^Research Group for Marine Geochemistry (ICBM-MPI Bridging Group), Institute for Chemistry and Biology of the Marine Environment, Carl von Ossietzky University of OldenburgOldenburg, Germany

**Keywords:** dissolved organic matter, fluorescence, absorbance, EEM-PARAFAC, ultrahigh resolution mass spectrometry, Florida coastal Everglades, subtropical wetland

## Abstract

Optical properties are easy-to-measure proxies for dissolved organic matter (DOM) composition, source, and reactivity. However, the molecular signature of DOM associated with such optical parameters remains poorly defined. The Florida coastal Everglades is a subtropical wetland with diverse vegetation (e.g., sawgrass prairies, mangrove forests, seagrass meadows) and DOM sources (e.g., terrestrial, microbial, and marine). As such, the Everglades is an excellent model system from which to draw samples of diverse origin and composition to allow classically-defined optical properties to be linked to molecular properties of the DOM pool. We characterized a suite of seasonally- and spatially-collected DOM samples using optical measurements (EEM-PARAFAC, SUVA_254_, S_275−295_, S_350−400_, S_R_, FI, freshness index, and HIX) and ultrahigh resolution mass spectrometry (FTICR-MS). Spearman's rank correlations between FTICR-MS signal intensities of individual molecular formulae and optical properties determined which molecular formulae were associated with each PARAFAC component and optical index. The molecular families that tracked with the optical indices were generally in agreement with conventional biogeochemical interpretations. Therefore, although they represent only a small portion of the bulk DOM pool, absorbance, and fluorescence measurements appear to be appropriate proxies for the aquatic cycling of both optically-active and associated optically-inactive DOM in coastal wetlands.

## Introduction

Dissolved organic matter (DOM) is an integral component of aquatic systems. DOM is fundamentally involved in many environmental processes, such as the binding of metals (Haitzer et al., 2002), transport of pollutants (Schwarzenbach et al., 2003), attenuation of light (Morris et al., 1995), and cycling of nutrients (Opsahl and Benner, 1997). DOM can also be chemically-altered via photoreactive (Spencer et al., 2009; Stubbins et al., 2010) and biodegradative (Spencer et al., 2015) processing. The composition of DOM is a reflection of both original source material and the degradative processing it undergoes once released in the aquatic environment. Elucidating the biogeochemical structure of DOM is key to understanding its dynamics and ultimate fate in aquatic ecosystems.

Optical spectroscopic techniques (e.g., absorbance and fluorescence) are a quick and relatively inexpensive means for assessing DOM quality (Fellman et al., 2010; Coble et al., 2014). Optical properties have been established as efficiently-measured proxies for DOM source and reactivity (Stedmon et al., 2003; Jaffé et al., 2008; Hernes et al., 2009). For example, bulk DOM aromaticity has been correlated with specific UV absorbance (SUVA_254_; Weishaar et al., 2003) and the fluorescence index (FI) can reflect relative contributions of microbial- or terrestrially-derived DOM sources (McKnight et al., 2001; Cory and McKnight, 2005). Three-dimensional excitation emission matrices (EEMs) have been widely used for the fluorescence-based characterization of DOM (Coble et al., 1990; Coble, 1996; Fellman et al., 2010; Ishii and Boyer, 2012). Early studies employed a “peak-picking” method to track changes in the EEM topography and relate these changes to DOM biogeochemistry (Coble et al., 1990; Coble, 1996). Current studies commonly use EEMs combined with parallel factor analysis (PARAFAC) to assess the environmental dynamics of DOM in diverse aquatic ecosystems (Jaffé et al., 2014). PARAFAC is a multivariate modeling technique, which statistically breaks down the EEM topographic signal into individual fluorescent components and estimates the relative contribution of these extracted components to the total EEM fluorescence of each DOM sample (Stedmon et al., 2003; Cory and McKnight, 2005). Although generally informative of DOM source and reactivity, optical measurements can only provide information about the optically-active compounds (e.g., chromophores and fluorophores) which exist as part of the bulk DOM pool. As such, very little is known about the optically-inactive compounds that track with chromophores and fluorophores as they enter and are then processed within aquatic ecosystems.

Fourier transform ion cyclotron resonance mass spectrometry (FTICR-MS) offers detailed molecular-level information regarding the composition of DOM (Kujawinski, 2002; Dittmar and Koch, 2006; Sleighter and Hatcher, 2007; Dittmar and Paeng, 2009). Due to ultrahigh mass accuracy, FTICR-MS resolves complex DOM mixtures allowing elemental formulae to be assigned to individual mass spectral peaks. As such, FTICR mass spectra yield unique molecular “fingerprints” for DOM. Although formulae cannot inherently be linked with specific molecular structures, as each represents many possible isomeric arrangements, they can be categorized by compound class using elemental ratios to help summarize the mass spectral composition of DOM (Šantl-Temkiv et al., 2013). Recent studies have correlated FTICR-MS signal intensities of individual molecular formulae with optical properties to assess which molecular families track with different PARAFAC components and optical indices in a drinking water reservoir (Herzsprung et al., 2012), boreal lakes (Kellerman et al., 2015), and rivers (Stubbins et al., 2014), and the open ocean (Timko et al., 2015). However, such comparisons have yet to be carried out in coastal or estuarine environments.

The Florida coastal Everglades is a subtropical wetland with diverse vegetation (sawgrass prairies, mangrove forests, seagrass meadows), salinity (ranging from fresh to hypersaline), and DOM sources (terrestrial, microbial and marine). Wetland DOM dynamics are complex due to variable OM inputs, geomorphology, hydrology, primary production, and degradation processes (Qualls and Richardson, 2003; Larsen et al., 2010; Yamashita et al., 2010; Chen et al., 2013; Chen and Jaffé, 2014). Since the Everglades is an oligotrophic wetland system, DOM also plays a key role with regards to nutrient cycling, as most nitrogen (N) exists in the organic form (Boyer et al., 1997; Boyer, 2006), a feature which is detectable by FTICR-MS (Hertkorn et al., 2015). Due to its heterogeneity, the Everglades is an excellent model system for assessing how classically-defined optical properties are linked to molecular properties of this highly diverse DOM pool. In this study, a suite of seasonally- and spatially-collected DOM samples were characterized using optical measurements (EEM-PARAFAC, SUVA_254_, S_275−295_, S_350−400_, S_*R*_, FI, freshness index and HIX), and FTICR-MS. Spearman's rank correlations between molecular formulae assigned to mass spectral peak intensities and optical indices were obtained to determine which molecular families associated with different DOM optical properties. Molecular families which were found to be associated with multiple optical properties are also discussed.

## Materials and methods

### Sample location and collection

**Figure 1:**
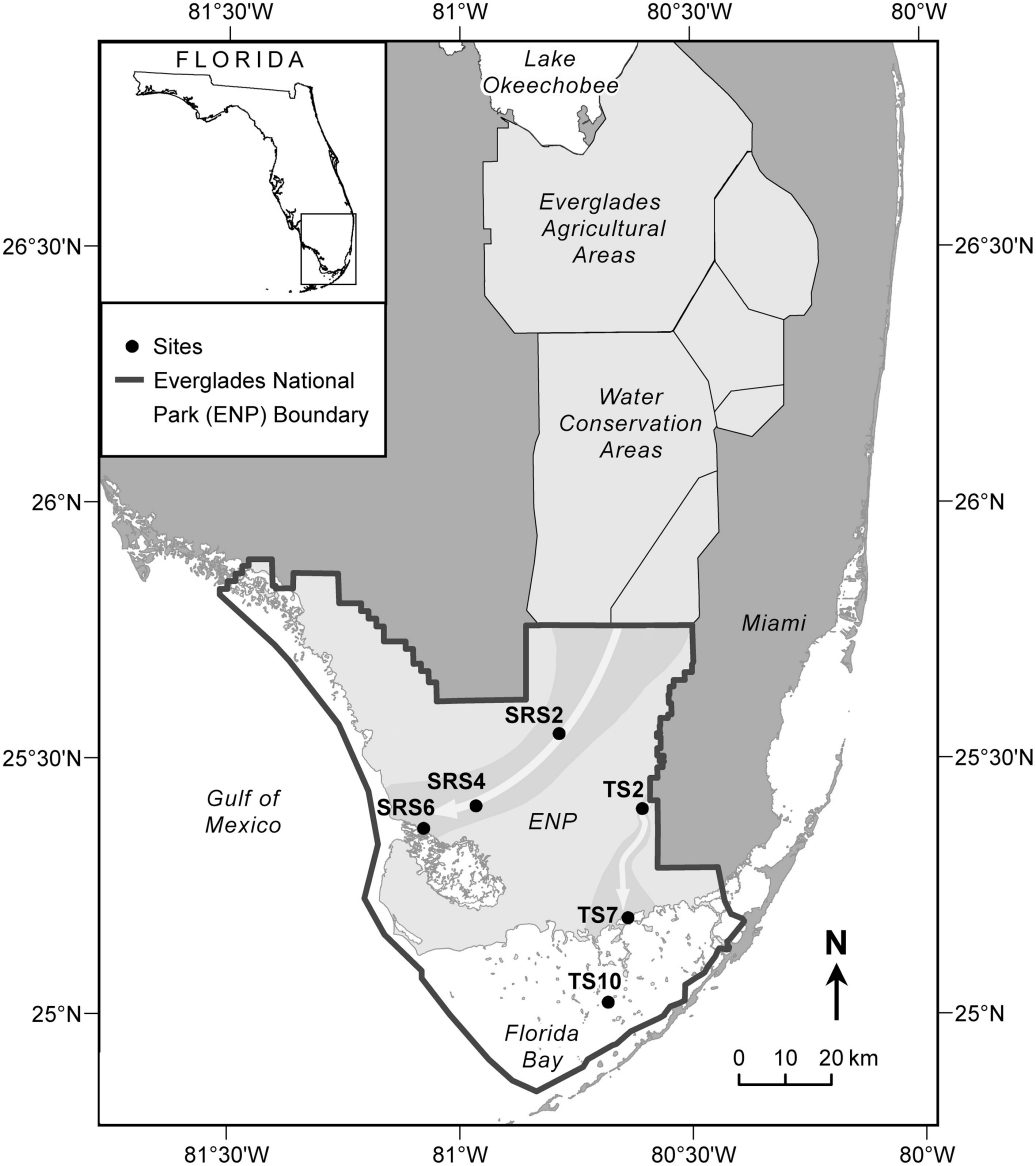
**Everglades sampling sites along Shark River Slough (SRS2, SRS4, SRS6) and Taylor Slough (TS2, TS7, TS10)**. Gray arrows show the flow of water along the Shark River Slough, which flows from the north to the southwest into the Gulf of Mexico, and Taylor Slough, which flows south into Florida Bay.

The Everglades is situated on the southern tip of the Florida peninsula where the regional climate is subtropical with distinct dry (November through April) and wet (May through October) seasons (Lodge, 2005). Vegetation cover is highly varied and its distribution is primarily driven by hydrology and salinity (Lodge, 2005). Sixty two surface water samples were collected monthly along two major flow paths between 2010 and 2011 at six sites (Figure 1) which are extensively monitored as part of the Florida Coastal Everglades long-term ecological research program (fce.lternet.edu). The sampling sites are described in brief, however further details can be found in Lodge (2005). Water drains through the Shark River Slough (SRS) from the north to the southwest into the Gulf of Mexico and is underlain with peat-based soil. In contrast, Taylor Slough (TS) drains in a general southern direction into Florida Bay, and is underlain primarily by marl-based soils. Sites SRS2 and TS2 are located in areas of freshwater marsh where the dominant vegetation is composed of emergent plants such as sawgrass and spikerush, and abundant periphyton mats. SRS2 and TS2 have distinctively different hydrological characteristics, with SRS2 featuring long hydroperiods (water depth and inundation time) compared to TS2 which undergoes routine dry-out during the dry season. SRS4, SRS6, and TS7 are estuarine sampling locations where flow paths are lined with mangrove forests. TS7, located at the mouth of the Taylor River, is highly influenced by hydrological changes between the wet and dry seasons when water is sourced primarily from inland freshwaters and Florida Bay, respectively. TS10 is a high salinity sampling location within Florida Bay, where seagrass meadows are the dominant form of vegetation. The current sample set includes monthly samples collected for 1 year (2010 to 2011) from SRS2 (*n* = 9), SRS4 (*n* = 12), SRS6 (*n* = 11), TS2 (*n* = 7), TS7 (*n* = 12), and TS10 (*n* = 10). Sites SRS2, SRS6, TS2, TS10 do not have samples representing all months. In these cases, the missing samples were either unavailable for analysis or not collected due to lack of water during the dry season. A complete list of sampling dates and exact locations are listed in Supplementary Table 1. Surface water samples were collected in pre-cleaned amber Nalgene bottles and stored on ice during transport back to the laboratory at Florida International University. Samples were filtered through pre-combusted GF/F filters (0.7 um) within 24 h of collection and stored in the dark at 4°C until further analysis.

### Analysis of dissolved organic carbon and optical measurements

DOC concentrations were measured for filtered samples using a Shimadzu TOC-V total organic carbon analyzer which employs the high-temperature catalytic combustion method (Stubbins and Dittmar, 2012). Absorbance spectra were collected (250–800 nm) using a Varian Cary 50 Bio UV-visible spectrophotometer and a quartz cuvette with a 1 cm path length. Absorbance at 254 nm was converted to Napierian absorbance coefficients (m^−1^; Hu et al., 2002) and used to measure chromophoric DOM content. Specific UV absorbance at 254 nm (SUVA_254_) is an indicator of DOM aromaticity and was defined as the absorbance at 254 nm (m^−1^) normalized to DOC (mg-C L^−1^; Weishaar et al., 2003). While the presence of iron has been shown to influence SUVA_254_ and other optical measurements (Poulin et al., 2014), the Everglades is underlain by limestone, severely limiting iron availability in this particular system. As such, SUVA_254_ corrections for iron were not carried out. Spectral slopes and the slope ratio (S_R_) have been used as proxies for DOM molecular weight, photo-alteration, bio-alteration (Helms et al., 2008), and lignin-normalized carbon yields (Spencer et al., 2010). Spectral slopes S_275−295_ and S_350−400_ were calculated by fitting a linear regression to the natural log-transformed absorbance between 275–295 and 350–400 nm, respectively. S_R_ was then simply calculated as the ratio of these spectral slopes (Helms et al., 2008).

**Figure 2:**
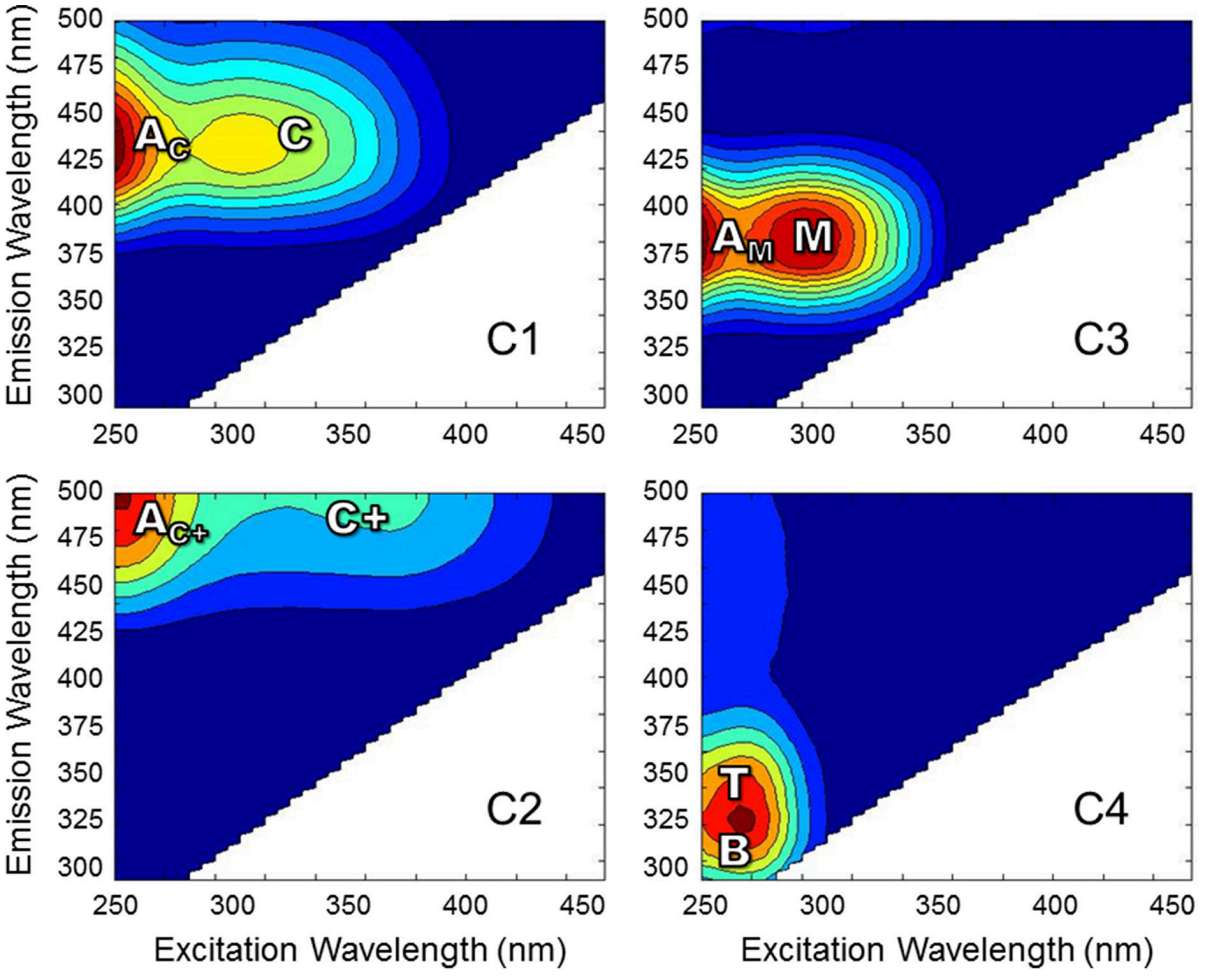
**EEMs of PARAFAC components for Everglades DOM**. Fluorescence spectra are labeled with conventional peaks A_C_, C, A_C+_, C+, A_M_, M, B, and T (defined in the text).

Excitation-emission matrices (EEMs) were obtained using a Horiba Jovin Yvon SPEX Fluoromax-3 spectrofluorometer equipped with a 150 W continuous output Xe arc lamp in a 1 cm quartz cuvette (Maie et al., 2006; Chen et al., 2010). Excitation and emission slit widths were 5.7 and 2 nm, respectively. Forty emission scans were acquired at excitation wavelengths (λ_ex_) 260–455 nm at 5 nm intervals. Emission wavelengths were scanned from λ_ex_ + 10 to λ_ex_ + 250 nm (i.e., between 250 and 705 nm) at 2 nm intervals. Individually-scanned spectra were then concatenated to generate EEMs. Post-acquisition data were blank-subtracted with Milli-Q water, absorbance data was used to correct for inner filter effects (McKnight et al., 2001) and fluorescence measurements were converted to quinine sulfate units. PARAFAC modeling was carried out using the Stedmon and Bro (2008) tutorial and accompanying Matlab code. EEMs from 57 of the 62 samples were used to develop the model and were normalized to a maximum intensity of 1 after being trimmed to remove the first and second order Rayleigh scatter. A four component model was validated using the split-half technique. EEM spectra were collected over a larger range than what is presented for the current PARAFAC model. Emission spectra collected at longer wavelengths tend to exhibit higher signal-to-noise ratios, thereby preventing model validation (Murphy et al., 2013). Therefore, EEMs for individual PARAFAC components shown in Figure 2 represent the validated spectral range. Upon validation, the model was applied to the original non-normalized EEMs in order to produce loadings that represented the original EEM intensities. In addition to PARAFAC, other fluorescence-derived indices were measured. The fluorescence index (FI) was measured as the ratio of emission intensity at 470 and 520 nm at excitation wavelength 370 nm (Cory and McKnight, 2005). The freshness index was calculated as the ratio of emission intensity at 380 nm to the maximum emission intensity between 420 and 435 nm at excitation wavelength 310 nm (Parlanti et al., 2000). The humification index (HIX) was calculated as the area under the emission spectra between 435 and 480 nm divided by the sum of peak areas 300 to 345 nm and 435 to 480 nm (Ohno, 2002).

### Dissolved organic matter extraction and mass spectrometry

Filtrates were acidified to pH 2 using concentrated HCl and the DOM was extracted following Dittmar et al. (2008). Briefly, DOM was isolated by solid phase extraction (SPE) by passing the acidified sample through a Varian Bond Elut PPL cartridge (5 g). SPE sorbent was first conditioned with MeOH and equilibrated with pH 2 Milli-Q water prior to DOM extraction. The filtrate passed through the cartridge via gravity and the sorbent was subsequently rinsed with pH 2 Milli-Q water for excess salt removal prior to drying under N_2_. DOM was then eluted with MeOH and stored in the dark at −20°C until mass spectral analysis.

Methanol PPL extracts were mixed with Milli-Q water (1:1 v/v) and continuously infused into the electrospray ionization (ESI) source of a Bruker Solarix 15 T FTICR-MS instrument (University of Oldenburg, Germany) at a flow rate of 120 uL h^−1^ in negative ion mode (500 scans). Mass spectra were calibrated using a reference mass list to achieve mass accuracies with an error less than 0.2 ppm. Molecular formulae consisting of C, H, O, N, S, and/or P were assigned to peaks with signal-to-noise ratios >5 according to published rules (Koch et al., 2007; Stubbins et al., 2010; Singer et al., 2012). Peak detection limits were standardized among samples by adjusting the dynamic range (DR) of each sample to that with the lowest DR (Stubbins et al., 2014). The DR was calculated for each sample as the average peak intensity of the highest 500 peaks divided by the signal-to-noise threshold intensity (average intensity of 10 lowest peaks). The standardized detection limit (SDL) was then set for each sample by dividing the average peak intensity of the 500 highest peaks by the lowest DR within the sample set. For each sample, peaks falling below the SDL were filled in with the SDL to prevent false negatives for samples with a low DR. ESI-FTICR-MS in negative ion mode primarily ionizes polar, organic compounds, which makes it the analytical method of choice for assessing the complex molecular composition of DOM mixtures (Kujawinski, 2002; Kim et al., 2003). However, it is well understood that ionization efficiencies are not equal among different compound classes. Therefore, the relative intensities of FTICR-MS peaks may not accurately reflect actual concentrations or be truly representative of the entire DOM pool.

Assigned molecular formulae were categorized by compound class according to elemental stoichiometries (Šantl-Temkiv et al., 2013). The modified aromaticity index (AI-mod) (Koch and Dittmar, 2006) classifies formulae as aromatic (polyphenols; 0.5 = AI-mod < 0.67) and condensed aromatic (black carbon; AI-mod = 0.67) formulae. Compound classes were further defined as highly unsaturated (AI-mod < 0.5, H/C < 1.5, O/C < 0.9), unsaturated aliphatics (1.5 = H/C < 2, O/C < 0.9, *N* = 0), saturated fatty acids (H/C = 2, (O/C < 0.9), sugars (O/C = 0.9), and peptides (1.5 = H/C < 2, O/C < 0.9, *N* < 0). Since an individual formulae could occur in multiple isomeric structures, these classifications only serve as a guide to the structures present within DOM.

### Spearman's rank correlations

Intensities for PARAFAC components were normalized to the sum of component fluorescence intensities within a sample. Mass spectral peak intensities were normalized to the sum intensity of all peaks within a sample. Pairwise Spearman's rank correlations (*r*) were then obtained between normalized PARAFAC components, optical indices (a_254_, SUVA_254_, S_275−295_, S_350−400_, S_R_, FI, freshness index, HIX), and normalized mass peak intensities. Correlations between molecular formulae and optical parameters were considered significant at the 99% confidence level (*p* < 0.01; Stubbins et al., 2014). Multivariate statistical analyses were carried out using JMP Pro 11 (SAS Institute Inc.).

## Results

### Dissolved organic carbon and optical measurements

DOC concentrations ranged from 3.3 to 20.4 mg-C L^−1^. DOC concentrations and salinity values for each sample are listed in Supplementary Table 1. Chromophoric DOM *a*_254_ ranged from 10 to 204 m^−1^ and SUVA_254_ from 0.7 to 4.9 mg-C L^−1^ m^−1^. The S_275−295_, S_350−400_, and S_*R*_ spanned ranges of 0.016 to 0.034 nm^−1^, 0.005 to 0.032 nm^−1^, and 0.68 to 3.15, respectively. Everglades FI ranged from 1.29 to 1.53, the freshness index from 0.45 to 0.87, and HIX from 1.6 to 15.1. A complete list of optical index values for all samples is detailed in Supplementary Table 2. PARAFAC modeling yielded four fluorescent components (C1–C4; Table 1; Figure 2). The loadings for each PARAFAC component are shown in Supplementary Figure 1. C1 is a “humic-like” component comprised of conventional peaks A_C_ and C (Figure 2). C2, another “humic-like” component, is shifted to longer emission wavelengths relative to C1 corresponding to conventional peak A_C+_ and C+ (Figure 2). C3 exhibits fluorescence within the region of conventional peaks A_M_ and M (Figure 2). C4 is a “protein-like” component, whose intensity maximum falls within the region of conventional peaks B and T (Figure 2). Prior EEM-PARAFAC studies on DOM in the greater Everglades (e.g., Chen et al., 2010; Yamashita et al., 2010) have employed an eight component PARAFAC model (from here on referred to as the FCE model where components are FCE1-FCE8; Chen et al., 2010). In the present study, EEMs were collected after sample dilution, resulting in a PARAFAC model that best fit only four components. Therefore, the eight component FCE model could not be directly applied to this particular data set. However, each of the 4 PARAFAC components employed in this study were statistically related to one or more components of the FCE model. At the 90% confidence level, C1 represented a mixture of model FCE1 + FCE3 (but not FCE1 alone), with likely contributions from FCE2 which exhibited similar emission patterns. However, the link between C1 and FCE2 could not be statistically validated. C2 represented FCE1 + FCE5, C3 represented FCE4 + FCE6 (but not FCE6 alone) and C4 represented FCE7. As such, the current 4 component model will be linked to the established FCE model in the discussion. Excitation and emission maxima for the current four component PARAFAC model are detailed in Table 1 where they are related to conventional peak assignments and FCE model components.

**Table 1:** **Excitation and emission maxima of PARAFAC components and their relation to conventional peaks as described by Coble et al. (2014)**.

**PARAFAC Component**	**Excitation/Emission Maxima (nm/nm)**	**Corresponding Conventional Peak(s)**	**Corresponding Component(s) from the FCE Model**	**Description**
C1	<260(ex)/425(em), 310(ex)/425(em)	A_C_/C	FCE1, FCE3	Ubiquitous humic-like Terrestrial humic-like
C2	260(ex)/>500(em), 370(ex)/>500(em)	A_C+_/C+	FCE1, FCE5	Ubiquitous humic-like Terrestrial humic-like
C3	<260(ex)/375(em), 300(ex)/375(em)	A_M_/M	FCE4, FCE6	Microbial humic-like
C4	275(ex)/320(em)	B/T	FCE7	Protein-like

*Components from the FCE PARAFAC model (Chen et al., 2010), which were statistically related to C1–C4 at the 90% confidence level, and their descriptions (from Yamashita et al., 2010) are also listed*.

### Ultrahigh resolution mass spectra

FTICR-MS allowed for the assignment of 6716 molecular formulae to resolved peaks across all DOM samples (Table 2). The formulae spanned a molecular mass range from 150 to 750 Da and most contained one or more N, S, and/or P atoms (*n* = 3756, 44% of intensity; Table 2). Of N-, S-, or P-containing formulae, CHON (*n* = 2494; 36% of total intensity) were the most abundant, followed by CHOS (*n* = 1531; 23% of total intensity; Table 2). Formulae with P were much less abundant (*n* = 251; 3% of total intensity; Table 2). CHO-only formulae contributed to less than half of the molecular formulae for this particular dataset (*n* = 2960, 45% of intensity; Table 2). Highly unsaturated formulae were ubiquitous and the dominant class of compounds for Everglades DOM (*n* = 3668, 53% of intensity; Table 2). Unsaturated aliphatics were also well represented (*n* = 909, 14% of intensity; Table 2). Aromatic compounds, such as black carbon (*n* = 587, 9% of intensity) and polyphenols (*n* = 1140, 18% of intensity) also had considerable contributions to the overall molecular signature (Table 2). Peptides (*n* = 275, 4% of intensity), saturated fatty acids (*n* = 100, 1% of intensity) and sugars (*n* = 37, 1% of intensity) contributed less to the molecular composition compared to other compound classes (Table 2).

**Table 2:** **Mean molecular mass, elemental groupings, and formula categorization of molecular formulae which correlated positively (***r*** > 0, ***p*** < 0.01) with PARAFAC components C1–C4**.

**Mean m/z (Da)**	**All Formulae**		**C1**		**C2**		**C3**		**C4**	
	**408**	**%**	**450**	**%**	**408**	**%**	**386**	**%**	**372**	**%**
Total no. formulae	6716	100	1980	29	2146	33	1761	26	1892	28
CHO only	2960	100	1122	37	1465	50	375	13	460	16
CHON	2494	100	842	34	238	10	986	39	952	38
CHOS	1531	100	55	3	462	32	528	34	628	41
CHOP	251	100	36	14	22	9	55	24	58	25
Contains N,S and/or P	3756	100	858	28	681	24	1386	45	1432	48
Black carbon	587	100	166	28	371	64	8	1	16	3
Polyphenols	1140	100	543	47	651	58	49	4	60	5
Highly unsaturated	3668	100	1214	33	1089	31	1069	28	1037	28
Unsaturated aliphatics	909	100	14	1	9	1	473	52	587	65
Saturated fatty acids	100	100	10	9	4	3	24	24	24	25
Sugars	37	100	11	29	10	28	3	9	2	6
Peptides	275	100	22	8	12	4	135	50	166	62

*Mean m/z is the intensity-weighted average mass (Da). Listed integers reflect the number of molecular formulae and percentages in parentheses represent the percent of peak intensity contributed by each classification of molecular formulae*.

## Discussion

### Dissolved organic carbon concentrations and optical indices

The Everglades landscape has diverse vegetation and its waters, which range from fresh to hypersaline, receive DOM inputs from both allochthonous and autochthonous sources (Chen et al., 2013). DOC concentrations and DOM optical properties vary considerably on both spatial and temporal scales. Everglades DOC concentrations have been reported to range from 3.6 to 28.0 mg-C L^−1^ (Yamashita et al., 2010; Chen et al., 2013; Ding et al., 2014) and SUVA_254_ values from 1.1 to 4.8 mg-C L^−1^ m^−1^ (Jaffé et al., 2008; Chen et al., 2013). The current sample set captured much of this variability in both DOC concentration and SUVA_254_ (Supplementary Tables 1, 2), the latter indicating that our Everglades DOM samples spanned a wide range in aromatic content (Weishaar et al., 2003; Stubbins et al., 2008). Spectral slopes and their ratio (S_R_) vary with the source, molecular weight and the photochemical and biological processing of DOM (Helms et al., 2008). Spectral slopes S_275−295_ and S_350−400_ have not been reported previously for the Everglades, however S_R_ values generally range from 0.7 to 9.0 from aromatic-rich swamp waters to the open ocean (Helms et al., 2008; Yamashita et al., 2010; Chen et al., 2013) but can exceed 13.0 in the high salinity waters of Florida Bay (Timko et al., 2014). The slope values reported here (Supplementary Table 2) cover a wide range of those reported for the Everglades and across gradients from highly terrigenous, black water swamp to open ocean DOM where S_275−295_ ranges from 0.013 to 0.036 nm^−1^, S_350−400_ from 0.008 to 0.019 nm^−1^, and S_R_ from 0.70 to 4.56 (Helms et al., 2008). Similarly, the measured FI for the current sample set (Supplementary Table 2) spans much of the variability reported for the Everglades which ranges from 1.28 to 1.47 (Yamashita et al., 2010). Across aquatic environments, FI values can range from 1.0 to 1.8 where low values indicate terrestrially-derived DOM and high values suggest DOM derived from microbial sources (Cory and McKnight, 2005; Jaffé et al., 2008). Although, HIX and freshness indices have not yet been reported for the Everglades, our dataset is representative of the possible range of HIX values from 4.5 to 16.0 (Singh et al., 2010) and freshness values from 0.4 to 0.9 (Huguet et al., 2010; Dixon et al., 2014) observed for other coastal systems which receive both terrestrial and marine DOM inputs. The diversity of DOM optical quality within the Everglades makes it an excellent model system from which to draw samples to allow classically defined optical properties to be linked to the molecular properties of the DOM pool. Based upon the above, the sample set analyzed here captured a wealth of this variability.

### PARAFAC components

Four PARAFAC components were assigned to the dataset (Table 1; Figure 2; Supplementary Figure 1). Their nomenclature is indicative of their proportional contribution to total fluorescence, which decreased from C1 to C4. C1 exhibited a primary peak at <260 nm(ex)/425 nm(em) and a secondary peak at 310 nm(ex)/425(em; Table 1; Figure 2). The primary peak falls in the region of the A_C_ peak and the secondary peak is within the region of the C peak as defined by the classical EEMs nomenclature (Stedmon et al., 2003; Fellman et al., 2010; Coble et al., 2014). Peaks A_C_ and C are typically described as representing terrigenous, high molecular weight, and aromatic DOM as they are commonly identified in forested and wetland environments (Fellman et al., 2010). C1 likely represents a mixture of “humic-like” components FCE1, FCE2, and FCE3 for the eight component model established by Chen et al. (2010; Table 1), where FCE1 is typically the most abundant fluorescent contributor (Chen et al., 2013). FCE2 is mainly derived from soil OM oxidation in the northern Everglades (Yamashita et al., 2010). FCE2 also represents fluorophores which are resistant to photo-degradation, while FCE3 has been suggested to be a photo-intermediate (Chen and Jaffé, 2014). As such, C1 in the current study likely represents the primary “humic-like,” soil-derived components in Everglades DOM. C2 exhibited a peak at 260 nm(ex)/>500 nm(em) with a shoulder peak at 370 nm(em) (Table 1; Figure 2) which has been observed in other terrestrially-dominated systems (e.g., Stedmon et al., 2003; Santín et al., 2009; Chen et al., 2010; Stubbins et al., 2014). This long wavelength peak has been referred to as the A_C+_/C+ peak and as being associated with older, terrestrial, or soil-derived organic material (Coble et al., 2014). C2 is similar to FCE1 + FCE5, both of which are terrestrially-derived, humic-like components and usually exhibit very similar environmental trends in the Everglades (Yamashita et al., 2010; Chen et al., 2013). C3 consisted of a primary peak at <260 nm(ex)/375 nm(em) and a secondary peak at 300 nm(em)/375 nm(em; Table 1; Figure 2). The peaks associated with C3 fall within the region of classical peaks A_M_ and M, which have been commonly found in marine surface waters and associated with microbial, freshly-produced and/or biolabile DOM (Coble et al., 1998, 2014; Fellman et al., 2010). C3 is also closely associated with FCE4 + FCE6, which have both been characterized as microbial humic-like components (Chen et al., 2010; Yamashita et al., 2010). FCE6 has also been shown to be highly photo-reactive (Chen and Jaffé, 2014). The C4 peak exhibited an intensity maximum at 275 nm(ex)/320 nm(em; Table 1; Figure 2). C4 is commonly referred to as a “protein-like” component (Fellman et al., 2010), and has been correlated with total hydrolysable amino acids (Yamashita and Tanoue, 2003). However, small nitrogen-free aromatic monomers have also been shown to fluoresce within the same region (Maie et al., 2007; Hernes et al., 2009). C4 is also well correlated with FCE7, which exhibits tyrosine-like fluorescence and has higher contributions to the fluorescence signature in areas of the Everglades where periphyton mats or seagrasses are abundant (Maie et al., 2012; Chen et al., 2013).

### Associations between PARAFAC components and molecular families

**Figure 3:**
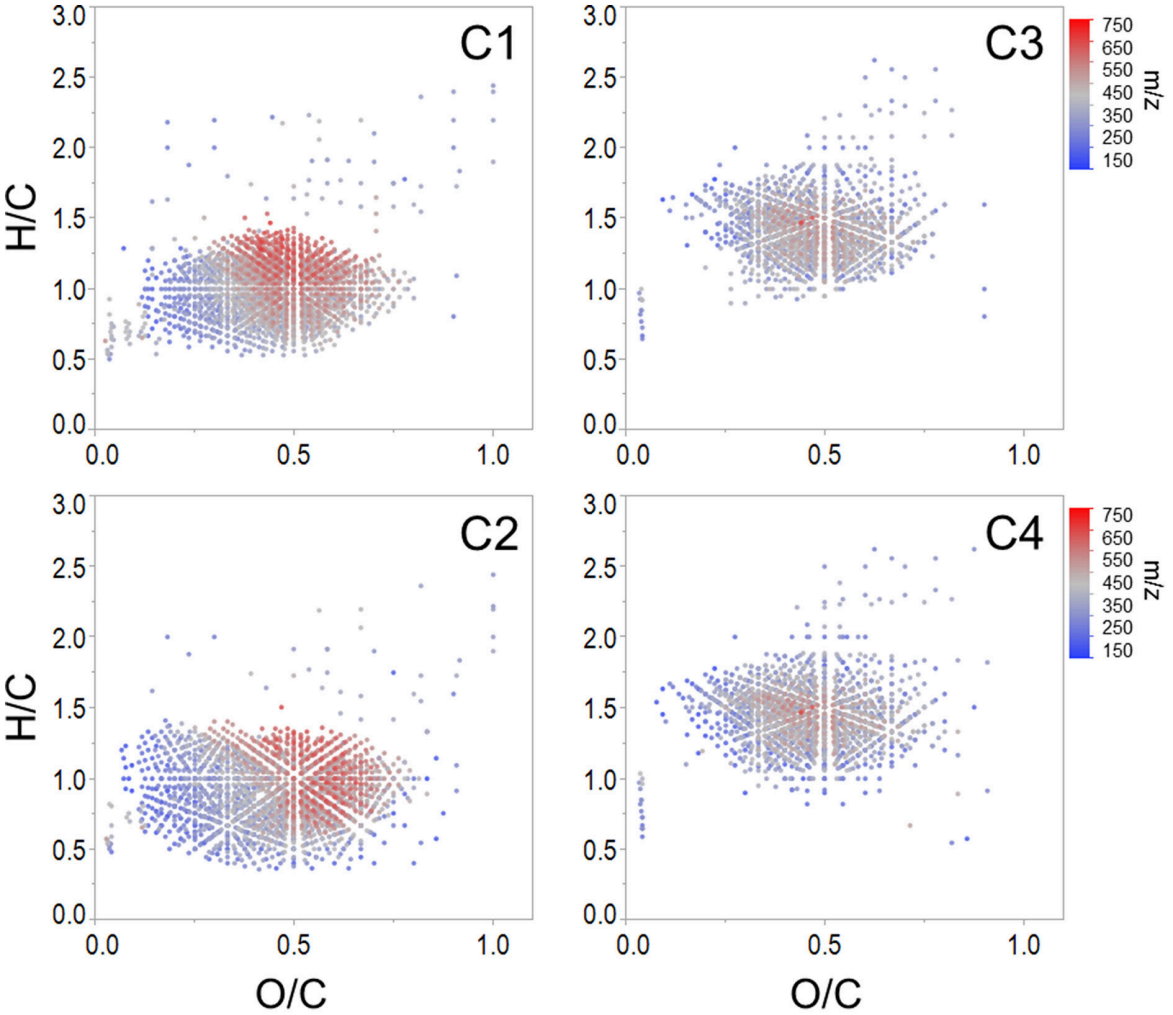
**Van Krevelen distributions of molecular formulae positively correlated with each Everglades PARAFAC component (corresponding EEMs are shown in Figure 2)**.

Spearman's rank correlations between normalized mass spectral peak intensities and normalized fluorescence intensities were carried out in order to elucidate groups of compounds associated with each PARAFAC component. The majority of assigned formulae tracked positively with one or more PARAFAC component (*n* = 5497, 82% of intensity), which suggests that C1 through C4 represented >80% of the molecular composition under the specific preparative and analytical conditions described for this study (i.e., PPL extraction followed by ESI-FTICR-MS analysis). Where EEM spectra are used to display the topographical distributions of fluorescence data, the molecular character of DOM revealed by FTICR-MS is facilitated by van Krevelen diagrams, where individual formulas are graphed according to their H/C and O/C ratios (Kim et al., 2003). Van Krevelen plots of molecular formulae associated with each PARAFAC component are shown in Figure 3. Additional van Krevelen distributions of each molecular grouping separated by N and S content are shown in Supplementary Figures 2–5.

As noted above, C1 (conventional peaks A_C_ and C; FCE1 + FCE3) and C2 (conventional peak A_C+_/C+; FCE1 + FCE5) represent “humic-like” components, which within the Everglades system are most likely terrestrially-sourced. Although C1 and C2 were both associated with high molecular weight, aromatic carbon-rich DOM (Table 2), individual formulae associated with these components spanned a wide range of masses, with small numbers of low molecular weight, aliphatic compounds also tracking with the “humic-like” fluorophores. Humic substances have been described to consist of supramolecular assemblies of smaller, heterogeneous molecules held together by weak dispersive forces or cation bridging (Piccolo, 2001; Simpson et al., 2002; Romera-Castillo et al., 2014). As such, the covariation of optically active (e.g., aromatic) and inactive (e.g., aliphatic) formulae may result from physical or chemical associations between these DOM components in natural waters. Optical measurements are a reflection of both the inherent optical properties of DOM compounds and how they functionally interact with one another. For example, DOM can participate in charge transfer (Sharpless and Blough, 2014) and interact with iron (Poulin et al., 2014), thereby altering the cumulative absorbent and/or fluorescent behavior of DOM. As such, DOM optical measurements may be better described as emergent properties of the total functionality and molecular interactions among all organic and inorganic constituents within a natural water sample (Stubbins et al., 2014).

C1 and C2 were broadly correlated with similar molecular families (Figure 3), however several notable distinctions exist. C1 was enriched in both CHO (*n* = 1122, 37% of intensity) and CHON (*n* = 842, 34% of intensity) formulae with considerable contributions from aromatic compounds, including black carbon and polyphenols (*n* = 166, 28% of intensity and *n* = 543, 47% of intensity, respectively; Table 2). C1 is a ubiquitous “humic-like” fluorophore which has been associated with terrestrial OM sources (Yamashita et al., 2010). In the Everglades, significant contributions of such “humic-like” components originate from soil OM oxidation throughout the system, primarily from the northern-most region of the Everglades Agricultural Area (EAA; Figure 1; Yamashita et al., 2010). Wildfires are common in the Everglades, and dissolved black carbon has been reported at relatively elevated levels compared to other locations globally (Jaffé et al., 2013; Ding et al., 2014), the routine burning of massive amounts of sugarcane in the EAA can serve as an additional source of highly aromatic pyrogenic DOM to the region. The CHON formulae associated with C1 are mainly aromatic, which may include dissolved black nitrogen (Wagner et al., 2015a) or other heterocyclic nitrogen compounds (Hertkorn et al., 2015). Enrichments in CHON formulae have been previously linked to anthropogenic land use (Wagner et al., 2015b). Therefore, C1 could indicate some degree of DOM input from the upstream EAA. Molecular formulae associated with C2 exhibited greater degrees of aromaticity compared to C1, with increased contributions from black carbon (*n* = 371, 64% of intensity) and polyphenols (*n* = 651, 58% of intensity; Table 2). In contrast to C1, formulae associated with C2 were relatively depleted in N, but enriched in CHOS formulae (*n* = 462, 32% of intensity; Table 2; Supplementary Figure 3). Although the aquatic cycling of dissolved organic sulfur in the Everglades is not well understood, subsurface peat soils can become enriched in sulfur-containing organic matter (Bates et al., 1998). Enrichments in CHOS formulae, likely resulting from the early diagenetic sulfurization of DOM, have been observed in soil and sediment pore waters of other marine-influenced systems (Schmidt et al., 2009). Such S-containing formulae have been previously reported for Everglades DOM (Hertkorn et al., 2015). Therefore, C2 may represent soil-derived or highly degraded DOM due to high S content and high relative contributions from biorefractory compound classes such as black carbon and other aromatics (Table 2).

Formulae associated with C3 and C4 shared very similar molecular compositions characterized by relatively low average molecular masses (386 Da and 372 Da, respectively) with increased contributions from N-, P-, or S-containing formulae, unsaturated aliphatics and peptides (Table 2, Supplementary Figures 4, 5). These components exhibited fluorescence at shorter wavelengths compared to C1 and C2, which may reflect an enrichment in aliphatics and lower molecular weight compounds from microbially-sourced DOM (Fellman et al., 2010). It was initially surprising to observe such a significant overlap between the molecular families associated with both C3 and C4 (Table 2; Figure 3). However, C3 and C4 were strongly correlated to one another for this dataset (*r* = 0.66, *p* < 0.001), and similar PARAFAC components for the FCE model (FCE4, FCE6, FCE7; Chen et al., 2010) have been observed to share similar spatial distributions throughout the Everglades landscape (Yamashita et al., 2010; Maie et al., 2012). Such similarities between C3 and C4 suggest common DOM sources (e.g., seagrasses, periphyton mats, primary productivity, or aquatic plants) and/or processing (e.g., bioavailability). Similar associations between conventional peaks M, B, and T and molecular formulae that are enriched in N, S, or P and aliphatic carbon have been observed previously across boreal river systems (Stubbins et al., 2014). In all cases, the PARAFAC components were associated with molecular formulas and characteristics are representative of their presumed origins and adequately describe their utility as proxies for bulk DOM characteristics.

### Associations between non-PARAFAC optical indices and molecular families

**Figure 4:**
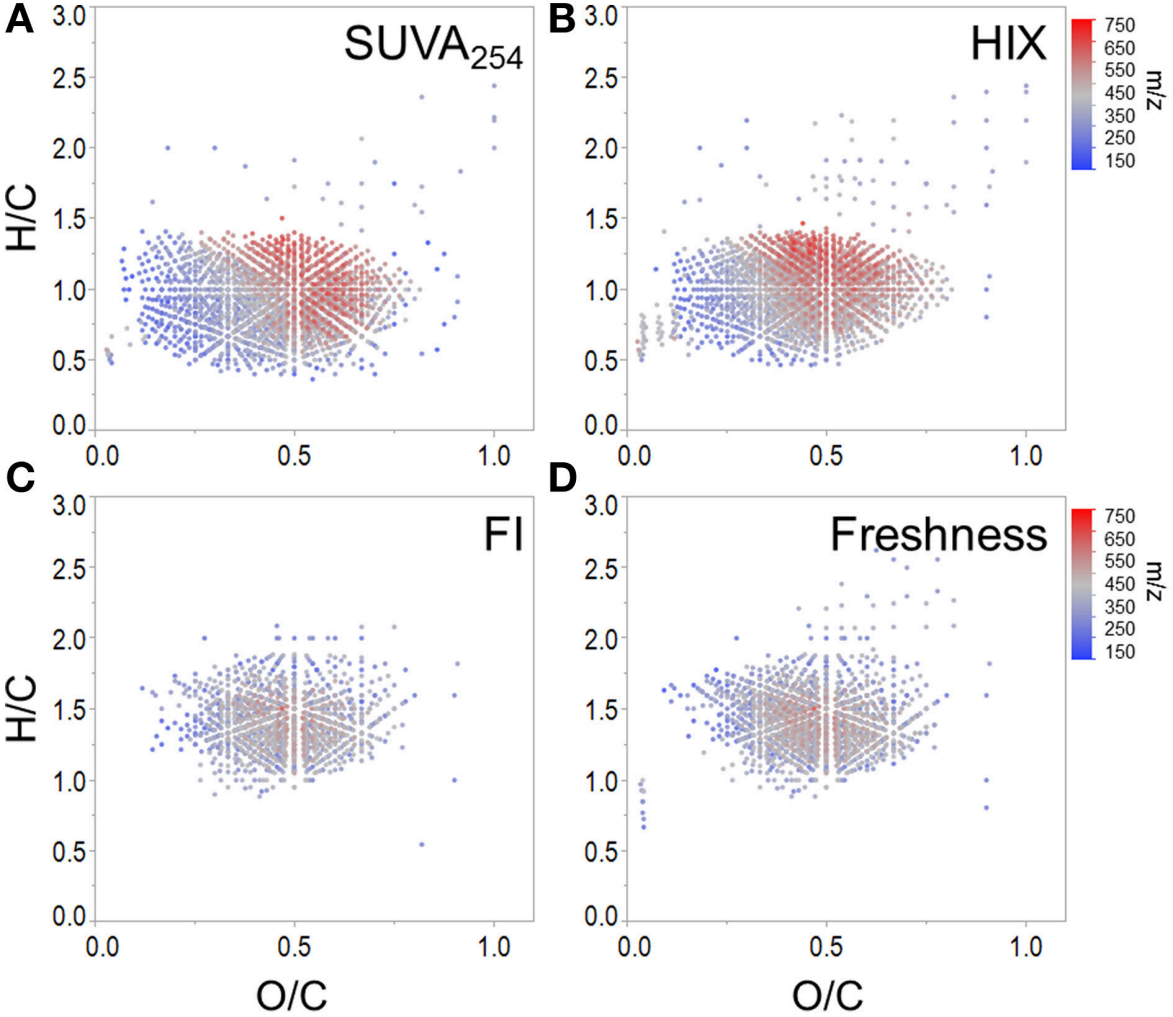
**Molecular formulae positively correlated with (A) SUVA_254_, (B) HIX, (C) FI, and (D) freshness index**.

**Figure 5:**
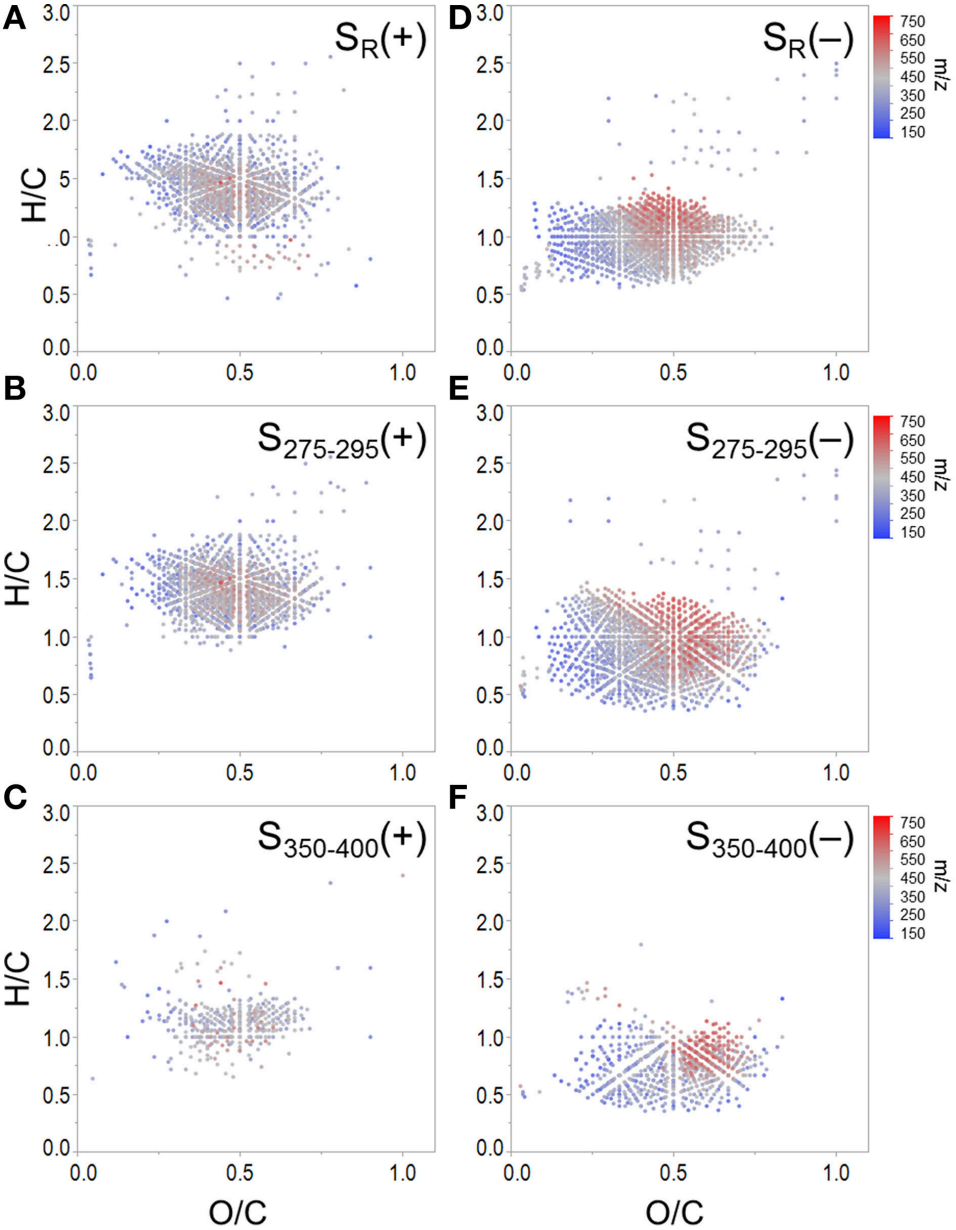
**Molecular formulae positively correlated with (A) S_R_, (B) S_275−295_ and (C) S_350−400_, and negatively correlated with (D) S_R_, (E) S_275−295_, and (F) S_350−400_**. Panels **(B)** and **(C)** show formulae assigned to mass spectral peaks which become more abundant as spectral slopes became steeper. Panels **(E)** and **(F)** show formulae assigned to mass spectral peaks which become more abundant as spectral slopes became shallower.

To expand on recent literature which primarily focused on how molecular formulae covaried with PARAFAC components alone (Stubbins et al., 2014), we also present formula groups associated with other commonly-employed optical indices. Measured values for all optical indices (SUVA_254_, S_275−295_, S_350−400_, S_R_, FI, freshness index, HIX) are listed in Supplementary Table 2. Spearman's correlations between ranked optical indices and normalized mass spectral peak intensities were obtained to identify molecular formulae associated with each index (*r* > 0, *p* < 0.01). The molecular composition of formulae positively correlated with each index is summarized in Table 3 and van Krevelen distributions are shown in Figures 4, 5. Detailed van Krevelen distributions of molecular formulae, separated by N and S content, associated with individual optical indices can be found in Supplementary Figures 6–12.

**Table 3:** **Mean molecular mass, elemental groupings, and formula categorization of molecular formulae which correlated positively (***r*** > 0, ***p*** < 0.01) with optical indices FI, freshness index, HIX, SUVA_254_, S_275−295_, S_350−400_, and S_R_**.

**Mean m/z (Da)**	**All Formulae**		**FI**		**Freshness**		**HIX**		**SUVA**_**254**_		**S**_**275**__**−**__**295**_		**S**_**350**__**−**__**400**_		**S**_*****R*****_	
	**408**	**%**	**383**	**%**	**385**	**%**	**450**	**%**	**413**	**%**	**382**	**%**	**407**	**%**	**379**	**%**
Total no. formulae	6716	100	1447	21	1869	27	2170	32	2162	33	1711	25	360	5	1635	24
CHO only	2960	100	125	4	362	12	1341	45	1429	49	320	11	35	1	372	13
CHON	2494	100	925	37	1016	40	683	28	388	16	912	36	312	12	734	29
CHOS	1531	100	530	34	641	42	195	13	359	25	599	39	9	1	634	41
CHOP	251	100	31	14	55	24	43	17	16	7	42	18	8	3	42	18
Contains N,S and/or P	3756	100	1322	44	1507	50	829	28	733	26	1391	46	325	10	1263	42
Black carbon	587	100	6	1	8	1	181	30	324	56	8	1	8	1	10	2
Polyphenols	1140	100	42	4	50	4	575	50	675	60	49	4	56	5	52	5
Highly unsaturated	3668	100	942	25	1129	30	1355	37	1135	32	1078	29	274	7	954	26
Unsaturated aliphatics	909	100	301	34	503	56	10	1	7	1	447	50	17	2	487	54
Saturated fatty acids	100	100	3	3	23	23	9	8	2	2	17	17	2	2	16	16
Sugars	37	100	3	9	4	12	13	36	8	22	2	6	3	8	1	3
Peptides	275	100	150	56	152	57	27	9	11	4	110	41	0	0	115	43

*Mean m/z is the intensity-weighted average mass (Da). Listed integers reflect the number of molecular formulae and percentages in parentheses represent the percent of peak intensity contributed by each classification of molecular formulae*.

SUVA_254_ increases with the chromophoric nature and degree of aromaticity of DOM (Weishaar et al., 2003). HIX has been shown to increase with the microbial processing of DOM (Wickland et al., 2007) and has been used to describe the degree of DOM humification (Zsolnay et al., 1999; Ohno, 2002). Optical indices SUVA_254_ and HIX were similarly effective in tracking a terrestrially-derived group of compounds enriched in aromatic formulae and depleted N, P, and S content (Table 3, Figures 4A,B). Black carbon compounds tracked most closely with SUVA_254_, whereas less condensed aromatic structures, such as polyphenols were more broadly associated with both HIX and SUVA_254_ (Table 3). Highly aromatic groups of DOM, as indicated by high *a*_254_ and SUVA_254_, can be photoreactive and degrade quickly when exposed to sunlight (Stubbins et al., 2008, 2010; Spencer et al., 2009; Stubbins and Dittmar, 2015). In addition, dissolved black carbon, as measured by chemo-oxidation methods, has been shown to be preferentially photodegraded relative to bulk DOM (Stubbins et al., 2012; Wagner and Jaffé, 2015). In this respect, the group of molecular formulae which tracked with SUVA_254_ and HIX in the Everglades (Figures 4A,B) overlap with those identified as photolabile in other studies (Kujawinski et al., 2004; Gonsior et al., 2009; Stubbins et al., 2010). As such, the covariation of formulae between these optical indices could be driven by both DOM source (e.g., soils or microbially degraded organic matter) and degradative processing (e.g., photodegradation). FI, initially put forth by McKnight et al. (2001), has been used to assess relative inputs from microbial vs. terrestrial precursor OM, and the freshness index estimates the relative proportion of recently-produced DOM (Parlanti et al., 2000; Wilson and Xenopoulous, 2009; Huguet et al., 2010). Both FI and the freshness index broadly represent autochthonous pools of DOM associated with microbial activity or exhibiting high degrees of biolability and were found to significantly correlate with one another for this particular dataset (*r* = 0.79, *p* < 0.001). As such, similar molecular families, enriched in biolabile aliphatic formulae (Spencer et al., 2015), were commonly associated with these indices (Table 3, Figure 4B). Previous work suggests that, in the Everglades, high FI/low SUVA_254_ values are indicative of tidal/marine or microbial DOM inputs and low FI/high SUVA_254_ values are indicative of freshwater marsh or mangrove-derived DOM inputs (Chen et al., 2013), which is consistent with the molecular trends observed here.

Spectral slopes and slope ratios derived from absorbance data have been related to DOM molecular weight, and the degree of CDOM photo- and bio-alteration (Helms et al., 2008). Van Krevelen distributions of molecular formulae positively and negatively correlated with S_R_, S_275−295_, and S_350−400_ are shown in Figure 5. S_R_ has been used as a proxy for DOM molecular weight, with higher S_R_ being indicative of lower molecular weight (Helms et al., 2008). Therefore, it is of interest that the group of formulae positively associated with S_R_ had, on average, relatively low masses (379 Da, Table 3; Figure 5A). Conversely, the group of formulae found to negatively associate with S_R_ had, on average, relatively high molecular masses (430 Da, Figure 5D). A considerable number of aliphatic molecular formulae (*n* = 1375) were positively correlated with both S_275−295_ and S_R_ (Table 3; Figures 5A,B), however a relatively unique molecular family was positively correlated with S_350−400_ (Figure 5C). Shallower spectral slopes across both wavelength ranges were generally associated with aromatic-rich, terrigenous DOM (e.g., low H/C, high molecular weight, more aromatic), but the molecular associations of S_275−295_ and S_350−400_ indicate that they may track with different pools of photo-labile, terrestrially-derived material (Figures 5E,F). Formulae associated with shallower S_275−295_ (Figure 5E) fall within the same van Krevelen regions as those which were positively correlated with SUVA_254_, HIX and C2, indicating that S_275−295_ provides similar information to these other indices. However, formulae which were negatively correlated with S_350−400_ occupy a unique region of van Krevelen space (Figure 5F) and were more oxidized (weighted average O/C = 0.50) and more aromatic (weighted average AI-mod = 0.56) than formulae negatively correlated with S_275−295_ (O/C = 0.45, AI-mod = 0.50; Figure 5E). Such highly oxidized, aromatic pools of DOM associated with S_350−400_ could be indicative of quinone-type moieties derived from terrestrial sources. A shallow spectral slope in the 350 to 400 nm range is indicative of high absorbance at long wavelengths (i.e., approaching 400 nm), which in turn indicates low energy transitions (Planck, 1901). The lower energy, longer wavelength absorbance of CDOM has been posited to arise from intra-molecular charge transfer facilitated by the presence of electron donor and acceptor groups within DOM molecules (Del Vecchio and Blough, 2004). The aromatic moieties that act as donors (e.g., polyhydroxylated aromatics, phenols, or indoles) and acceptors (e.g., quinones) all have oxygen containing substituent groups and are proposed to derive from the partial oxygenation of common terrigenous precursors such as lignin, tannins, and melanins. Therefore, perhaps the oxygen-enriched DOM compounds associated with shallow S_350−400_ (Figure 5F) are indicative of moieties which facilitate charge transfer.

### Assessing molecular covariance among PARAFAC components and optical indices

**Figure 6:**
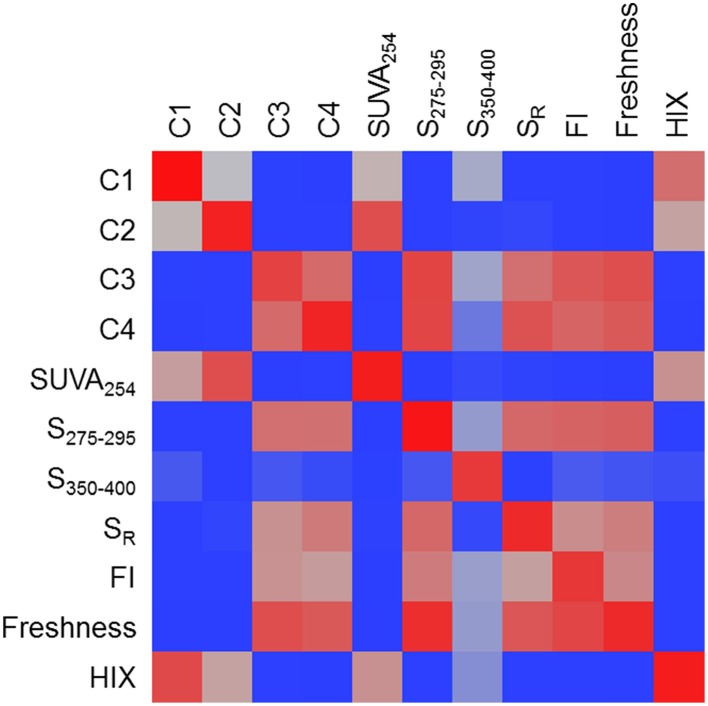
**A heat map of the number of molecular formulae which tracked positively with multiple PARAFAC components and/or optical indices**. Blocks are colored along a gradient from blue (few or no shared formulae) to red (many shared formulae). Red blocks indicate that very similar molecular families are associated with two optical properties.

**Figure 7:**
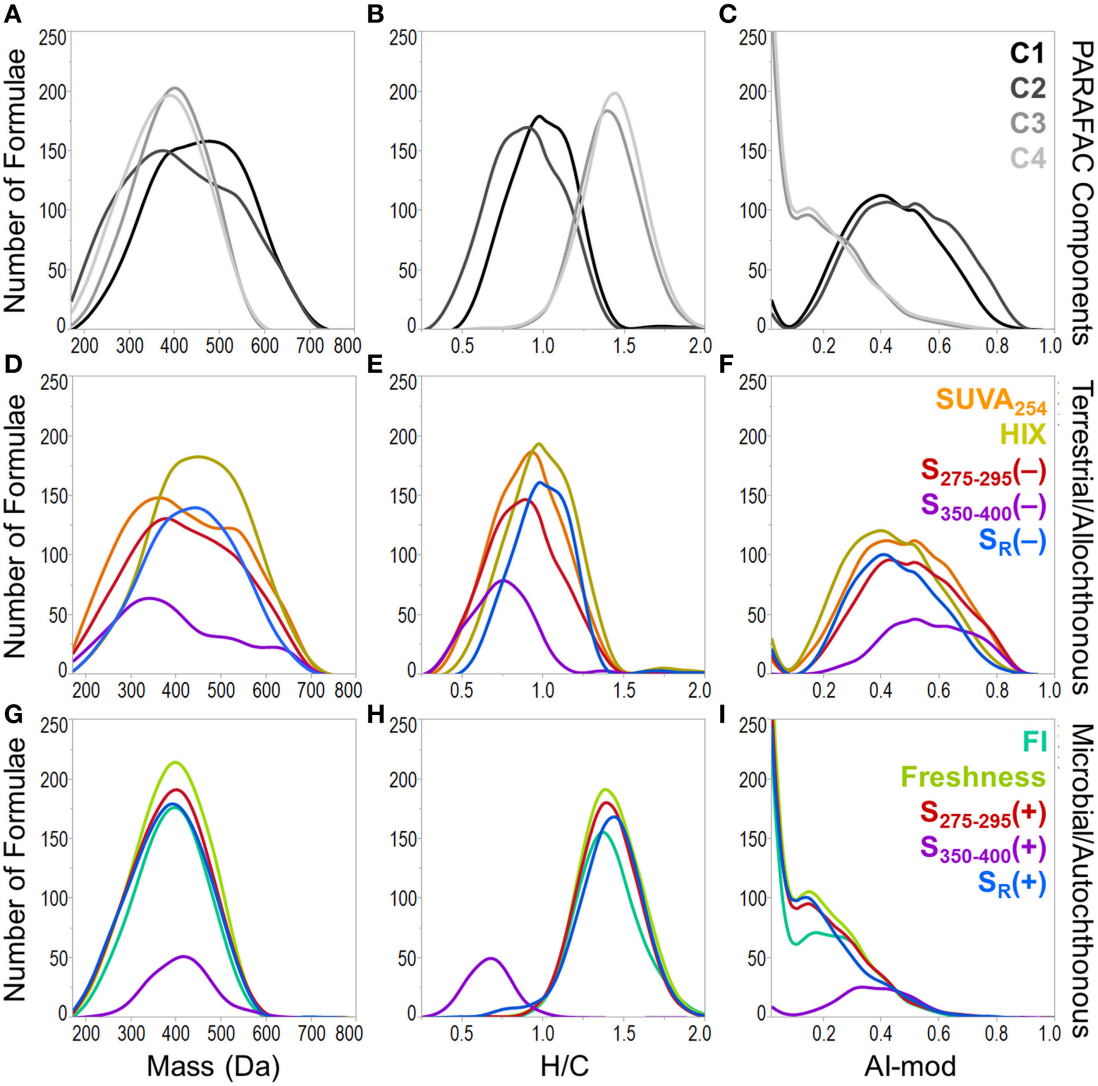
**Molecular mass (first column), H/C ratio (second column) and modified aromaticity index (AI-mod; third column) distributions of formulae associated with optical properties**. Plots **(A–C)** show the distributions of formulae which positively correlated with PARAFAC components. Plots **(D–F)** show the distributions for optical properties which track with allochthonous or terrestrial-type DOM (formulae positively correlated with SUVA_254_, HIX and formulae negatively correlated with S_275−295_, S_350−400_, and S_R_). Plots **(G–I)** show the distributions for optical properties which track with autochthonous or microbial-type DOM (formulae positively correlated with FI, freshness index, S_275−295_, S_350−400_, and S_R_).

A heat map was constructed to visually describe the number of molecular formulae shared among PARAFAC components and/or optical indices (Figure 6). Blocks are colored along a gradient from blue (few or no shared formulae) to red (many shared formulae; Figure 6). Line plots showing the mass, H/C and AI-mod distribution of molecular formulae associated with PARAFAC components and the other optical indices are shown in Figure 7. The distinct difference in molecular compositions associated with C1 and C2 vs. C3 and C4 is clearly apparent (Figures 7A–C). All non-PARAFAC optical indices were categorized into one of two groups: those which generally tracked with aromatic-rich, allochthonous/terrestrial DOM (Figures 7D–F) and those which generally tracked with autochthonous/microbial DOM (Figures 7G–I). The line distribution plots allow for a more detailed comparison among molecular families associated with optical properties. For example, a group of high molecular weight, terrestrially-derived formulae found to be associated with C1 also tracked similarly with HIX (Figure 7). C2 and SUVA_254_ were both correlated with the most aromatic-enriched pools of molecular formulae (Figure 7). Similarities in molecular character between C3 and C4 have been described above, however FI, freshness index, S_295−275_(+) and S_R_(+) seem to be equally as effective in tracking the same pool of aliphatic-enriched compounds as the two PARAFAC components (Figures 6, 7). S_350−400_ resolved a distinct group of molecular formulae (described above) which was not associated with any PARAFAC component (Figures 6, 7D–F). This suggests that, for the current dataset, absorbance data (e.g., SUVA_254_, S_295−275_, and S_R_) can provide nearly equivalent information regarding the molecular composition of DOM as PARAFAC modeling. However, it is important to note that such an observation can only be made for this particular suite of DOM samples using a four component PARAFAC model, which only offers coarse resolution of the EEM topography. The extraction of additional components may reveal more refined associations between molecular formulae and identified fluorophores for specific aquatic systems (e.g., see Stubbins et al., 2014; Kellerman et al., 2015).

## Conclusions

Conventional biogeochemical interpretations of PARAFAC components and other optical indices were generally in agreement with the bulk DOM molecular composition of formulae with which they were associated for the Everglades system. These patterns were also fairly consistent with previous research assessing optical and molecular linkages in other terrestrial aquatic systems (Stubbins et al., 2014; Kellerman et al., 2015). Allochthonous and autochthonous molecular families, which tracked with SUVA_254_/HIX and FI/freshness index, respectively (Figure 4), were in agreement with molecular associations put forth by Kellerman et al. (2015). Regarding PARAFAC components, a significant overlap in formulae associated with both conventional A_M_/M and B/T peaks (i.e., characterized by low molecular weight, aliphatic, high N content) was also observed in high latitude lakes (Kellerman et al., 2015) and boreal rivers (Stubbins et al., 2014), which suggests that “microbial humic-like” and “protein-like” fluorescence may serve as equally reliable trackers of autochthonous DOM across diverse aquatic environments. Although, “humic-like” components were generally associated with terrestrially-sourced, aromatic and high molecular weight formulae in lakes (Kellerman et al., 2015), rivers (Stubbins et al., 2014) and wetlands (this study), notable differences were evident with regards to N content. For example, formulae associated with conventional A_C_/C peaks in lacustrine (Kellerman et al., 2015) and wetland (this study) environments had considerable contributions from CHON formulae, whereas molecular signatures associated with similar fluorescence peaks identified in boreal rivers were much more depleted in N (Stubbins et al., 2014). Such molecular discrepancies may be driven by fundamental differences in source OM (e.g., primary productivity in lakes and wetlands vs. higher plants and soils in terrestrial river systems) or DOM processing. Stubbins et al. (2014) have suggested that the conventional A_C_ peak may serve as a proxy for black carbon due to the enrichment in condensed aromatic formulae which tracked with that particular component in boreal rivers. However, black carbon formulae tracked more closely with PARAFAC components exhibiting the A_C+_ peak in both the Everglades and lake environments (Kellerman et al., 2015). Aquatic systems can vary significantly with regards to DOM source, functionality and reactivity. As such, researchers interested in correlating molecular composition with chromophores and fluorophores are encouraged to establish such relationships for their own systems and assess how formulae associated with such optical parameters compare across different environments.

Optical measurements, which reflect both the light-absorbing and light-emitting molecular components of DOM, are indicative of both the inherent optical properties of DOM compounds and how they interact with one another under specific environmental conditions. As such, DOM absorbance and fluorescence measurements are better described as emergent properties of the physico-chemical interactions among all organic and inorganic constituents which exist in the dissolved phase of a natural water sample. In the current study, the molecular families which tracked with optical indices were generally in agreement with conventional biogeochemical interpretations. In most cases, the optical indices broadly represented either terrestrial/allochthonous or microbial/autochthonous groups of DOM formulae. Although absorbance and fluorescence measurements represent a relatively small portion of bulk DOM pools, they have been validated here as informative proxies for the aquatic cycling of both optically-active and associated optically-inactive DOM in coastal wetlands and other aquatic environments.

## Author contributions

KC collected optical data and built the PARAFAC model and TD collected FTICR-MS data. SW interpreted the data and conducted statistical analyses with comments and suggestions from AS, RJ, and KC. SW wrote the manuscript with critical reviews and input from AS, RJ, KC, and TD.

### Conflict of interest statement

The authors declare that the research was conducted in the absence of any commercial or financial relationships that could be construed as a potential conflict of interest.
