# Supplementary material for: Associations Between the Molecular and Optical Properties of Dissolved Organic Matter in the Florida Everglades, a Model Coastal Wetland System
Source: Front Chem. 2015 Nov 25;3:66. doi: 10.3389/fchem.2015.00066 (PMC4658450; doi:10.3389/fchem.2015.00066)
Supplement: Supplementary file 1 [file DataSheet1.DOCX]

*Supplementary Material*

**Associations between the molecular and optical properties of dissolved organic matter in the Florida Everglades, a model coastal wetland system**

Sasha Wagner*^1^, Rudolf Jaffé^2^, Kaelin Cawley^3^, Thorsten Dittmar^4^, Aron Stubbins^1^

^1^Skidaway Institute of Oceanography, Marine Sciences Department, University of Georgia, Savannah, Georgia, USA

^2^Department of Chemistry and Biochemistry, Southeast Environmental Research Center (SERC), Florida International University, Miami, Florida, USA

^3^Department of Civil, Environmental and Architectural Engineering, Institute of Arctic and Alpine Research (INSTAAR), University of Colorado at Boulder, Boulder, Colorado, USA

^4^Research Group for Marine Geochemistry (ICBM-MPI Bridging Group), University of Oldenburg, Institute for Chemistry and Biology of the Marine Environment (ICBM), Oldenburg, Germany

***Correspondence:** Dr. Sasha Wagner

Skidaway Institute of Oceanography

Marine Sciences Department

University of Georgia

10 Ocean Science Circle

Savannah, GA, 31411, USA

sasha.wagner@skio.uga.edu

**1 Supplementary Tables and Figures**

**1.1 Supplementary Figures**


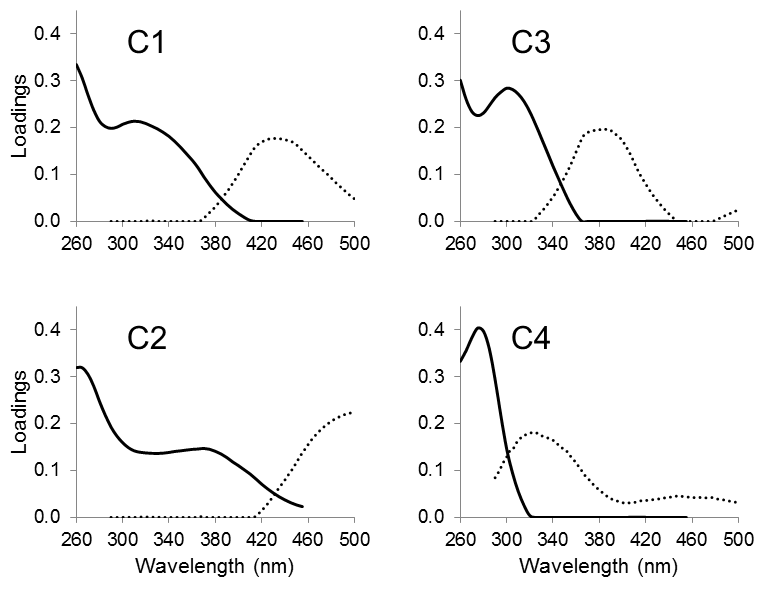
**Supplementary Figure 1.** PARAFAC loadings for components 1 through 4 (C1 – C4). Solid lines represent excitation spectra and dotted lines represent emission spectra.


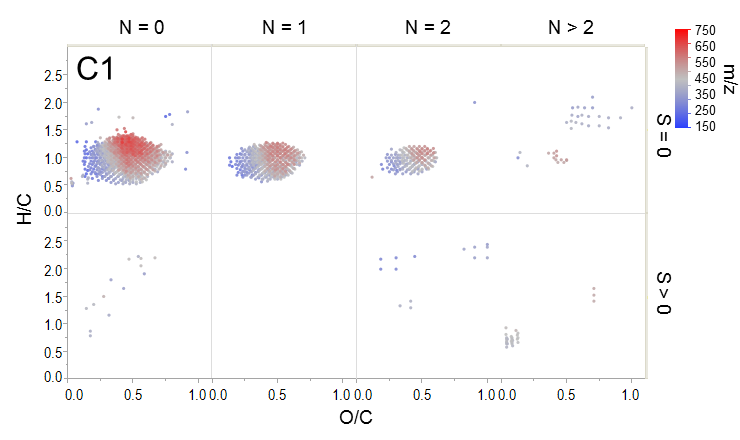
**Supplementary Figure 2.** Molecular formulae positively correlated (r > 0, p < 0.01) with PARAFAC component C1. Formulae are plotted into separate van Krevelen diagrams according to the number of N atoms (0, 1, 2 or >2) and S atoms (0 or >0).


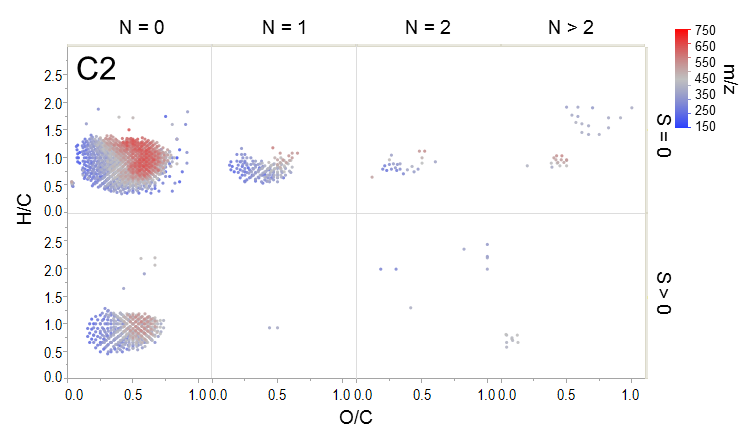


**Supplementary Figure 3.** Molecular formulae positively correlated (r > 0, p < 0.01) with PARAFAC component C2. Formulae are plotted into separate van Krevelen diagrams according to the number of N atoms (0, 1, 2 or >2) and S atoms (0 or >0).


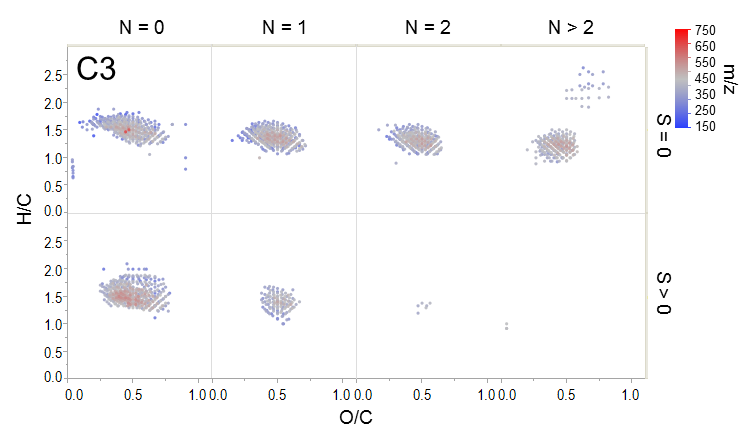


**Supplementary Figure 4.** Molecular formulae positively correlated (r > 0, p < 0.01) with PARAFAC component C3. Formulae are plotted into separate van Krevelen diagrams according to the number of N atoms (0, 1, 2 or >2) and S atoms (0 or >0).


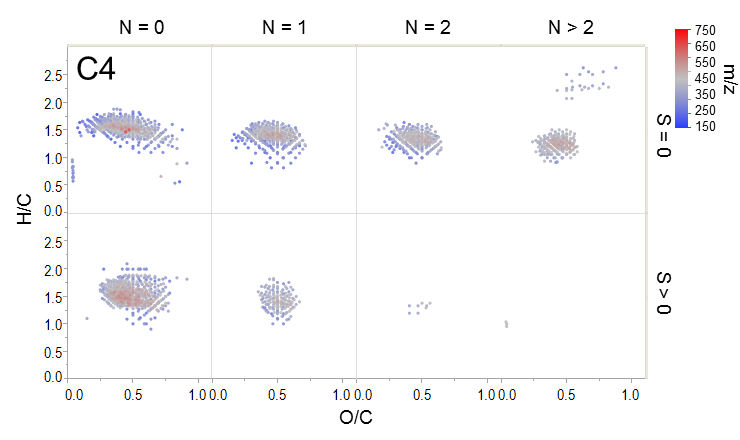


**Supplementary Figure 5.** Molecular formulae positively correlated (r > 0, p < 0.01) with PARAFAC component C4. Formulae are plotted into separate van Krevelen diagrams according to the number of N atoms (0, 1, 2 or >2) and S atoms (0 or >0).


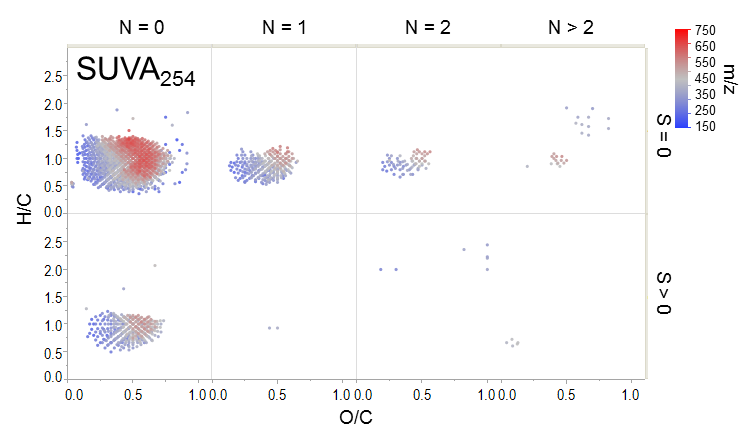


**Supplementary Figure 6.** Molecular formulae positively correlated (r > 0, p < 0.01) with specific UV absorbance (SUVA_254_). Formulae are plotted into separate van Krevelen diagrams according to the number of N atoms (0, 1, 2 or >2) and S atoms (0 or >0).


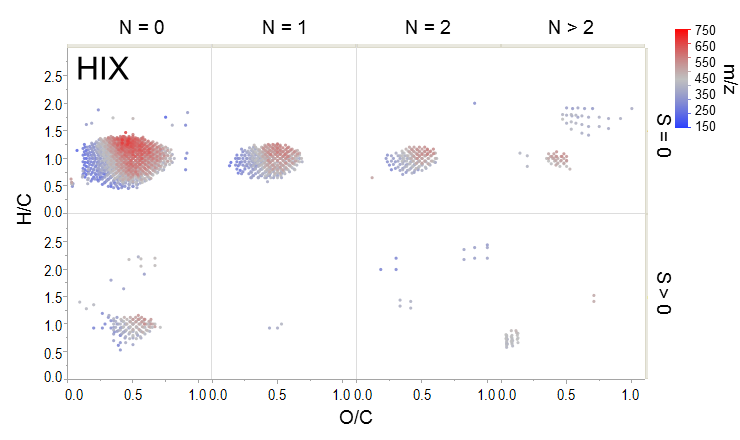


**Supplementary Figure 7.** Molecular formulae positively correlated (r > 0, p < 0.01) with the humification index (HIX). Formulae are plotted into separate van Krevelen diagrams according to the number of N atoms (0, 1, 2 or >2) and S atoms (0 or >0).


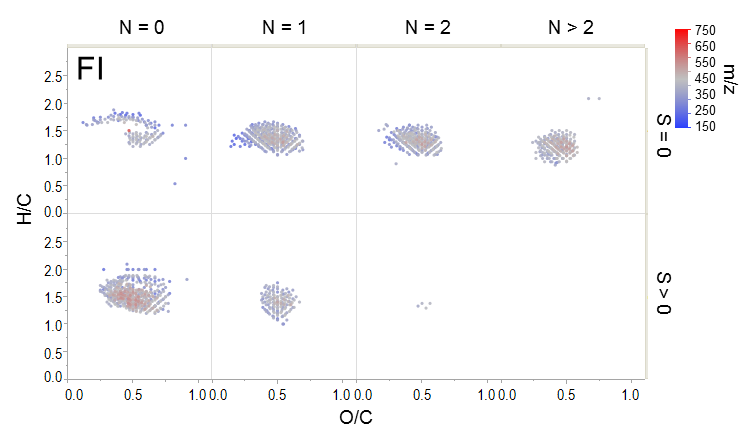


**Supplementary Figure 8.** Molecular formulae positively correlated (r > 0, p < 0.01) with the fluorescence index (FI). Formulae are plotted into separate van Krevelen diagrams according to the number of N atoms (0, 1, 2 or >2) and S atoms (0 or >0).


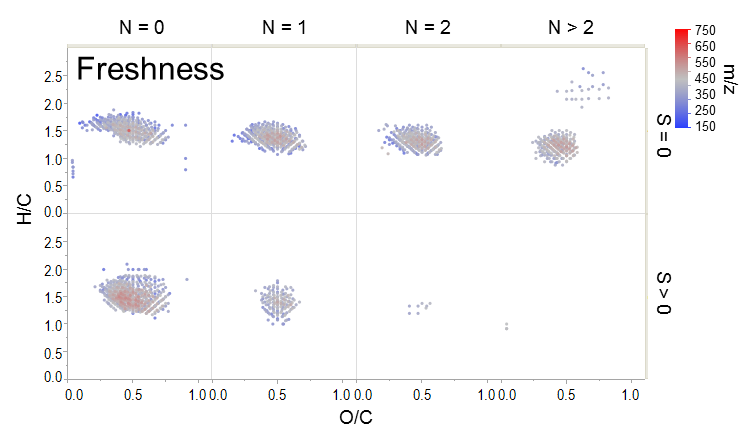


**Supplementary Figure 9.** Molecular formulae positively correlated (r > 0, p < 0.01) with the freshness index. Formulae are plotted into separate van Krevelen diagrams according to the number of N atoms (0, 1, 2 or >2) and S atoms (0 or >0).


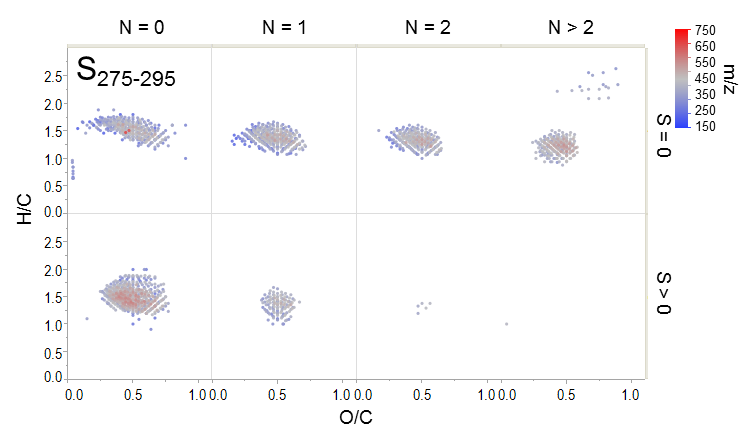


**Supplementary Figure 10.** Molecular formulae positively correlated (r > 0, p < 0.01) with spectral slope (S_275-295_). Formulae are plotted into separate van Krevelen diagrams according to the number of N atoms (0, 1, 2 or >2) and S atoms (0 or >0).


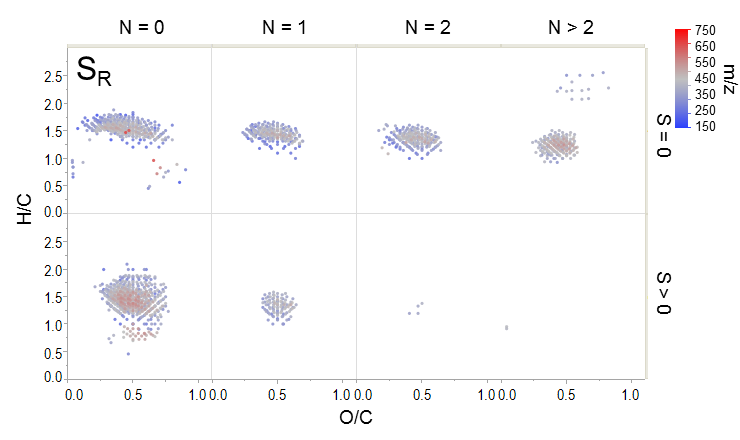


**Supplementary Figure 11.** Molecular formulae positively correlated (r > 0, p < 0.01) with the slope ratio (S_R_). Formulae are plotted into separate van Krevelen diagrams according to the number of N atoms (0, 1, 2 or >2) and S atoms (0 or >0).


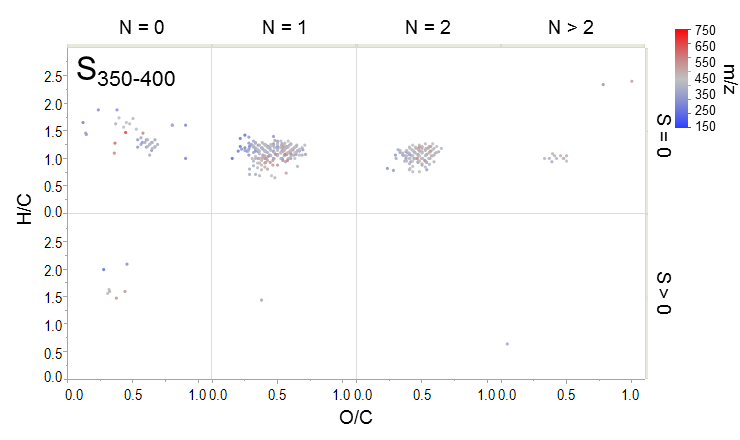


**Supplementary Figure 12.** Molecular formulae positively correlated (r > 0, p < 0.01) with spectral slope (S_350-400_). Formulae are plotted into separate van Krevelen diagrams according to the number of N atoms (0, 1, 2 or >2) and S atoms (0 or >0).


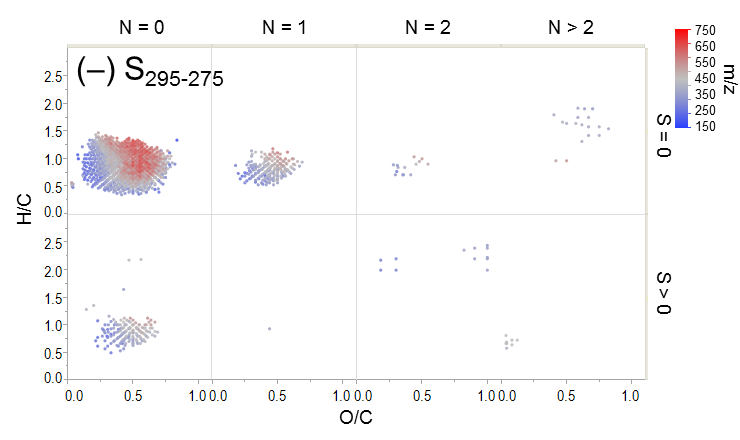


**Supplementary Figure 13.** Molecular formulae negatively correlated (r < 0, p < 0.01) with S_275-295_. Formulae are plotted into separate van Krevelen diagrams according to the number of N atoms (0, 1, 2 or >2) and S atoms (0 or >0).


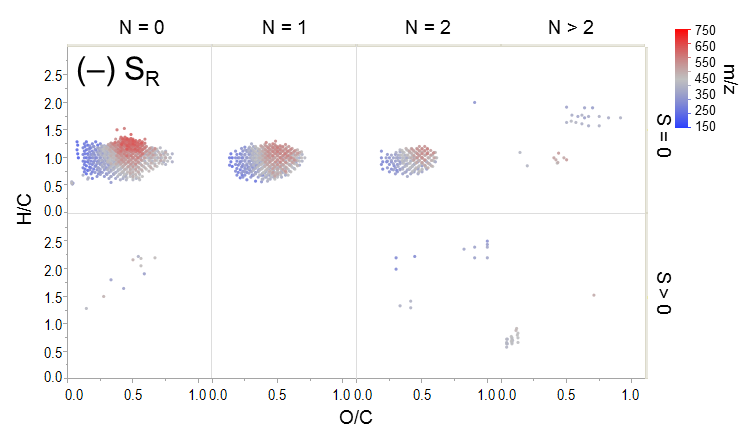


**Supplementary Figure 14.** Molecular formulae negatively correlated (r < 0, p < 0.01) with S_R_. Formulae are plotted into separate van Krevelen diagrams according to the number of N atoms (0, 1, 2 or >2) and S atoms (0 or >0).


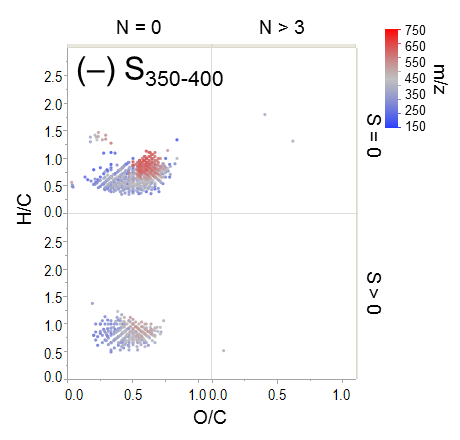


**Supplementary Figure 15.** Molecular formulae negatively correlated (r < 0, p < 0.01) with S_350-400_. Formulae are plotted into separate van Krevelen diagrams according to the number of N atoms (0, 1, 2 or >2) and S atoms (0 or >0).

**2.2 Supplementary Tables**

**Supplementary Table 1.** Dissolved organic carbon (DOC) concentrations, salinity and relative contribution of PARAFAC components for monthly samples collected along Shark River Slough (SRS2, SRS4, SRS6) and Taylor Slough (TS2, TS7, TS10). “NA” indicates that data was not available.

| Site | Year | Month | DOC (mg-C L^-1^) | Salinity (ppt) | C1 | C2 | C3 | C4 |
| --- | --- | --- | --- | --- | --- | --- | --- | --- |
| SRS2 | 2010 | July | 20.4 | 0.0 | 0.449 | 0.295 | 0.191 | 0.065 |
| SRS2 | 2010 | August | 13.4 | 0.0 | 0.418 | 0.315 | 0.166 | 0.101 |
| SRS2 | 2010 | September | 13.2 | 0.0 | 0.444 | 0.323 | 0.158 | 0.075 |
| SRS2 | 2010 | October | 14.4 | 0.0 | 0.446 | 0.325 | 0.162 | 0.068 |
| SRS2 | 2010 | November | 11.4 | 0.0 | 0.450 | 0.319 | 0.160 | 0.071 |
| SRS2 | 2010 | December | 17.9 | 0.0 | 0.445 | 0.325 | 0.166 | 0.064 |
| SRS2 | 2011 | January | 17.2 | 0.0 | 0.444 | 0.326 | 0.167 | 0.063 |
| SRS2 | 2011 | February | 13.9 | 0.0 | 0.446 | 0.325 | 0.165 | 0.064 |
| SRS2 | 2011 | March | 20.3 | 0.0 | 0.427 | 0.287 | 0.177 | 0.109 |
| SRS4 | 2010 | July | 18.8 | 1.1 | 0.441 | 0.315 | 0.180 | 0.063 |
| SRS4 | 2010 | August | 15.0 | 1.7 | 0.445 | 0.339 | 0.151 | 0.065 |
| SRS4 | 2010 | September | 13.4 | 1.6 | 0.445 | 0.346 | 0.152 | 0.056 |
| SRS4 | 2010 | October | 13.2 | 0.5 | 0.436 | 0.358 | 0.146 | 0.059 |
| SRS4 | 2010 | November | 13.3 | 2.1 | 0.431 | 0.343 | 0.157 | 0.069 |
| SRS4 | 2010 | December | 13.4 | 1.5 | 0.448 | 0.332 | 0.167 | 0.053 |
| SRS4 | 2011 | January | 13.6 | 3.6 | 0.431 | 0.338 | 0.164 | 0.067 |
| SRS4 | 2011 | February | 11.5 | 3.6 | 0.445 | 0.342 | 0.165 | 0.048 |
| SRS4 | 2011 | March | 11.8 | 9.2 | 0.436 | 0.348 | 0.165 | 0.052 |
| SRS4 | 2011 | April | 14.8 | 19.5 | 0.422 | 0.348 | 0.161 | 0.070 |
| SRS4 | 2011 | May | 14.3 | 19.2 | 0.433 | 0.367 | 0.162 | 0.039 |
| SRS4 | 2011 | June | 14.7 | 18.9 | NA | NA | NA | NA |
| SRS6 | 2010 | July | 11.0 | 24.1 | 0.424 | 0.337 | 0.188 | 0.051 |
| SRS6 | 2010 | August | 10.4 | 22.5 | 0.427 | 0.369 | 0.150 | 0.054 |
| SRS6 | 2010 | October | 10.9 | 12.0 | 0.423 | 0.364 | 0.150 | 0.063 |
| SRS6 | 2010 | November | 10.3 | 20.2 | 0.412 | 0.360 | 0.152 | 0.076 |
| SRS6 | 2010 | December | 5.9 | 27.7 | 0.413 | 0.348 | 0.170 | 0.069 |
| SRS6 | 2011 | January | 8.2 | 22.4 | 0.423 | 0.359 | 0.160 | 0.058 |
| SRS6 | 2011 | February | 5.8 | 24.3 | 0.421 | 0.365 | 0.160 | 0.054 |
| SRS6 | 2011 | March | 5.5 | 28.0 | 0.416 | 0.362 | 0.164 | 0.058 |
| SRS6 | 2011 | April | 5.7 | 33.0 | 0.405 | 0.355 | 0.171 | 0.069 |
| SRS6 | 2011 | May | 7.3 | 31.9 | NA | NA | NA | NA |
| SRS6 | 2011 | June | 7.5 | 32.2 | NA | NA | NA | NA |

**Supplementary Table 1.** (continued)

| Site | Year | Month | DOC (mg-C L^-1^) | Salinity (ppt) | C1 | C2 | C3 | C4 |
| --- | --- | --- | --- | --- | --- | --- | --- | --- |
| TS2 | 2010 | July | 10.3 | 0.0 | 0.410 | 0.250 | 0.204 | 0.136 |
| TS2 | 2010 | August | 7.9 | 0.0 | 0.438 | 0.269 | 0.176 | 0.117 |
| TS2 | 2010 | September | 7.9 | 0.0 | 0.436 | 0.265 | 0.185 | 0.114 |
| TS2 | 2010 | October | 8.2 | 0.0 | 0.423 | 0.295 | 0.179 | 0.103 |
| TS2 | 2010 | November | 7.0 | 0.0 | 0.447 | 0.262 | 0.208 | 0.084 |
| TS2 | 2010 | December | 5.3 | 0.0 | 0.442 | 0.330 | 0.180 | 0.048 |
| TS2 | 2011 | January | 5.4 | 0.0 | 0.442 | 0.336 | 0.177 | 0.044 |
| TS2 | 2011 | February | 3.3 | 0.0 | 0.441 | 0.335 | 0.179 | 0.045 |
| TS7 | 2010 | July | 19.2 | 8.9 | 0.419 | 0.292 | 0.195 | 0.093 |
| TS7 | 2010 | August | 16.3 | 15.5 | 0.419 | 0.339 | 0.156 | 0.087 |
| TS7 | 2010 | September | 20.4 | 2.6 | 0.440 | 0.362 | 0.134 | 0.064 |
| TS7 | 2010 | October | 11.3 | 0.6 | 0.420 | 0.382 | 0.124 | 0.074 |
| TS7 | 2010 | November | 10.2 | 4.0 | 0.408 | 0.347 | 0.147 | 0.098 |
| TS7 | 2010 | December | 8.5 | 5.4 | 0.412 | 0.331 | 0.163 | 0.094 |
| TS7 | 2011 | January | 8.6 | 8.2 | 0.391 | 0.297 | 0.175 | 0.136 |
| TS7 | 2011 | February | 6.7 | 3.6 | 0.400 | 0.320 | 0.163 | 0.117 |
| TS7 | 2011 | March | 9.5 | 20.6 | NA | NA | NA | NA |
| TS7 | 2011 | April | 9.5 | 24.5 | 0.339 | 0.252 | 0.203 | 0.205 |
| TS7 | 2011 | May | 11.0 | 31.2 | 0.355 | 0.288 | 0.196 | 0.160 |
| TS7 | 2011 | June | 10.2 | 38.6 | 0.338 | 0.242 | 0.210 | 0.211 |
| TS10 | 2010 | September | 7.1 | 40.1 | 0.325 | 0.207 | 0.217 | 0.251 |
| TS10 | 2010 | October | 6.6 | 28.3 | 0.325 | 0.220 | 0.208 | 0.247 |
| TS10 | 2010 | November | 5.9 | 30.4 | 0.324 | 0.218 | 0.206 | 0.251 |
| TS10 | 2010 | December | 7.5 | 28.7 | 0.319 | 0.203 | 0.202 | 0.276 |
| TS10 | 2011 | January | 8.2 | 31.7 | 0.363 | 0.222 | 0.217 | 0.198 |
| TS10 | 2011 | February | 6.3 | 33.3 | 0.356 | 0.218 | 0.217 | 0.209 |
| TS10 | 2011 | March | 5.1 | 35.6 | 0.346 | 0.215 | 0.220 | 0.219 |
| TS10 | 2011 | April | 4.7 | 40.4 | NA | NA | NA | NA |
| TS10 | 2011 | May | 6.4 | 41.7 | 0.273 | 0.166 | 0.188 | 0.373 |
| TS10 | 2011 | June | 3.6 | 40.4 | 0.300 | 0.226 | 0.183 | 0.292 |

**Supplementary Table 2.** Absorbance at 254nm (*a*_254_), specific UV absorbance at 254nm (SUVA_254_), spectral slopes (S_275-295_, S_350-400_), slope ratio (S_R_), fluorescence index (FI), freshness index and humification index (HIX) for monthly samples collected along Shark River Slough (SRS2, SRS4, SRS6) and Taylor Slough (TS2, TS7, TS10). “NA” indicates that data was not available.

| Site | Year | Month | *a*254 (m^-1^) | SUVA_254_ (mg-C L^-1^ m^-1^) | S_275-295_ (nm^-1^) | S_350-400_ (nm^-1^) | S_R_ | FI | Freshness Index | HIX |
| --- | --- | --- | --- | --- | --- | --- | --- | --- | --- | --- |
| SRS2 | 2010 | July | 141 | 3.0 | 0.019 | 0.019 | 0.97 | 1.34 | 0.62 | 10.2 |
| SRS2 | 2010 | August | 110 | 3.5 | 0.018 | 0.019 | 0.94 | 1.29 | 0.56 | 6.8 |
| SRS2 | 2010 | September | 105 | 3.5 | 0.018 | 0.019 | 0.96 | 1.33 | 0.53 | 7.7 |
| SRS2 | 2010 | October | 99 | 3.0 | 0.019 | 0.019 | 0.97 | 1.34 | 0.55 | 10.8 |
| SRS2 | 2010 | November | 82 | 3.1 | 0.017 | 0.019 | 0.88 | 1.35 | 0.55 | 6.9 |
| SRS2 | 2010 | December | 118 | 2.9 | 0.019 | 0.020 | 0.94 | 1.38 | 0.57 | 8.4 |
| SRS2 | 2011 | January | 127 | 3.2 | 0.020 | 0.021 | 0.93 | 1.38 | 0.58 | 7.4 |
| SRS2 | 2011 | February | 131 | 4.1 | 0.020 | 0.021 | 0.93 | 1.36 | 0.56 | 7.4 |
| SRS2 | 2011 | March | 194 | 4.1 | 0.017 | 0.018 | 0.97 | 1.41 | 0.61 | 4.3 |
| SRS4 | 2010 | July | 153 | 3.5 | 0.017 | 0.017 | 1.00 | 1.33 | 0.60 | 9.7 |
| SRS4 | 2010 | August | 131 | 3.8 | 0.017 | 0.016 | 1.01 | 1.33 | 0.51 | 7.7 |
| SRS4 | 2010 | September | 118 | 3.8 | 0.017 | 0.016 | 1.05 | 1.37 | 0.52 | 10.0 |
| SRS4 | 2010 | October | 111 | 3.7 | 0.017 | 0.017 | 1.02 | 1.33 | 0.52 | 10.4 |
| SRS4 | 2010 | November | 101 | 3.3 | 0.019 | 0.018 | 1.03 | 1.35 | 0.56 | 6.1 |
| SRS4 | 2010 | December | 106 | 3.4 | 0.019 | 0.019 | 1.03 | 1.39 | 0.58 | 11.3 |
| SRS4 | 2011 | January | 120 | 3.8 | 0.019 | 0.018 | 1.10 | 1.37 | 0.59 | 7.0 |
| SRS4 | 2011 | February | 125 | 4.7 | 0.019 | 0.019 | 1.01 | 1.40 | 0.58 | 10.6 |
| SRS4 | 2011 | March | 122 | 4.5 | 0.019 | 0.018 | 1.07 | 1.39 | 0.59 | 11.0 |
| SRS4 | 2011 | April | 23 | 0.7 | 0.020 | 0.029 | 0.69 | 1.39 | 0.59 | 9.5 |
| SRS4 | 2011 | May | 123 | 3.7 | 0.018 | 0.017 | 1.05 | 1.38 | 0.58 | 15.2 |
| SRS4 | 2011 | June | 147 | 4.3 | 0.017 | 0.015 | 1.14 | NA | NA | NA |
| SRS6 | 2010 | July | 91 | 3.6 | 0.018 | 0.016 | 1.09 | 1.37 | 0.65 | 10.3 |
| SRS6 | 2010 | August | 95 | 4.0 | 0.017 | 0.016 | 1.07 | 1.36 | 0.53 | 10.2 |
| SRS6 | 2010 | October | 99 | 3.9 | 0.018 | 0.017 | 1.05 | 1.35 | 0.53 | 8.1 |
| SRS6 | 2010 | November | 81 | 3.4 | 0.018 | 0.017 | 1.03 | 1.36 | 0.54 | 5.0 |
| SRS6 | 2010 | December | 50 | 3.7 | 0.020 | 0.018 | 1.11 | 1.36 | 0.60 | 8.7 |
| SRS6 | 2011 | January | 74 | 3.9 | 0.019 | 0.017 | 1.10 | 1.39 | 0.58 | 8.1 |
| SRS6 | 2011 | February | 65 | 4.9 | 0.019 | 0.019 | 1.00 | 1.36 | 0.57 | 8.0 |
| SRS6 | 2011 | March | 57 | 4.5 | 0.019 | 0.017 | 1.11 | 1.38 | 0.60 | 10.4 |
| SRS6 | 2011 | April | 35 | 2.6 | 0.021 | 0.024 | 0.86 | 1.38 | 0.63 | 9.4 |
| SRS6 | 2011 | May | 59 | 3.5 | 0.018 | 0.016 | 1.10 | NA | NA | NA |
| SRS6 | 2011 | June | 75 | 4.3 | 0.017 | 0.013 | 1.28 | NA | NA | NA |

**Supplementary Table 2.** (continued)

| Site | Year | Month | a254 (m^-1^) | SUVA254 (mg-C L^-1^ m^-1^) | S_275-295_ (nm^-1^) | S_350-400_ (nm^-1^) | S_R_ | FI | Freshness | HIX |
| --- | --- | --- | --- | --- | --- | --- | --- | --- | --- | --- |
| TS2 | 2010 | July | 56 | 2.4 | 0.020 | 0.019 | 1.02 | 1.39 | 0.69 | 5.2 |
| TS2 | 2010 | August | NA | NA | NA | NA | NA | 1.42 | 0.60 | 5.6 |
| TS2 | 2010 | September | 43 | 2.3 | 0.020 | 0.018 | 1.08 | 1.38 | 0.60 | 5.9 |
| TS2 | 2010 | October | 39 | 2.1 | 0.022 | 0.019 | 1.17 | 1.40 | 0.62 | 7.0 |
| TS2 | 2010 | November | 33 | 2.1 | 0.020 | 0.023 | 0.86 | 1.46 | 0.63 | 6.5 |
| TS2 | 2010 | December | 29 | 2.4 | 0.020 | 0.021 | 0.95 | 1.51 | 0.63 | 10.1 |
| TS2 | 2011 | January | 38 | 3.1 | 0.018 | 0.022 | 0.84 | 1.46 | 0.62 | 10.8 |
| TS2 | 2011 | February | 28 | 3.7 | 0.021 | 0.031 | 0.68 | 1.48 | 0.62 | 10.6 |
| TS7 | 2010 | July | 127 | 2.9 | 0.019 | 0.017 | 1.15 | 1.38 | 0.66 | 6.9 |
| TS7 | 2010 | August | 126 | 3.4 | 0.018 | 0.016 | 1.14 | 1.37 | 0.55 | 6.1 |
| TS7 | 2010 | September | 204 | 4.3 | 0.016 | 0.015 | 1.07 | 1.31 | 0.47 | 7.9 |
| TS7 | 2010 | October | 114 | 4.4 | 0.016 | 0.015 | 1.06 | 1.32 | 0.45 | 7.3 |
| TS7 | 2010 | November | 83 | 3.5 | 0.018 | 0.016 | 1.12 | 1.35 | 0.53 | 5.1 |
| TS7 | 2010 | December | 65 | 3.3 | 0.020 | 0.017 | 1.17 | 1.35 | 0.58 | 6.7 |
| TS7 | 2011 | January | 54 | 2.7 | 0.022 | 0.019 | 1.17 | 1.35 | 0.64 | 4.4 |
| TS7 | 2011 | February | 63 | 4.1 | 0.021 | 0.018 | 1.19 | 1.37 | 0.60 | 4.7 |
| TS7 | 2011 | March | NA | NA | NA | NA | NA | NA | NA | NA |
| TS7 | 2011 | April | 29 | 1.3 | 0.034 | NA | NA | 1.42 | 0.81 | 3.3 |
| TS7 | 2011 | May | 34 | 1.3 | 0.026 | 0.016 | 1.62 | 1.38 | 0.75 | 4.4 |
| TS7 | 2011 | June | 34 | 1.4 | 0.024 | 0.008 | 3.15 | 1.46 | 0.80 | 3.2 |
| TS10 | 2010 | September | 19 | 1.2 | 0.029 | 0.018 | 1.65 | 1.50 | 0.87 | 2.3 |
| TS10 | 2010 | October | 17 | 1.1 | 0.032 | 0.032 | 1.02 | 1.51 | 0.83 | 2.3 |
| TS10 | 2010 | November | 18 | 1.3 | 0.029 | 0.022 | 1.36 | 1.51 | 0.84 | 2.2 |
| TS10 | 2010 | December | 26 | 1.5 | 0.029 | 0.020 | 1.46 | 1.49 | 0.84 | 2.4 |
| TS10 | 2011 | January | 36 | 1.9 | 0.028 | 0.024 | 1.19 | 1.41 | 0.80 | 3.6 |
| TS10 | 2011 | February | 34 | 2.4 | 0.028 | 0.023 | 1.24 | 1.47 | 0.79 | 3.2 |
| TS10 | 2011 | March | 24 | 2.1 | 0.030 | 0.017 | 1.74 | 1.45 | 0.84 | 3.2 |
| TS10 | 2011 | April | 10 | 0.9 | 0.031 | NA | NA | NA | NA | NA |
| TS10 | 2011 | May | 18 | 1.3 | 0.028 | 0.020 | 1.39 | 1.48 | 0.86 | 1.6 |
| TS10 | 2011 | June | 15 | 1.9 | 0.017 | 0.005 | 3.04 | 1.53 | 0.82 | 2.4 |
